# Evaluation of *CD16*, *CD32*, *CD40*, and *CD152* polymorphisms in immune thrombocytopenia patients: a systematic review, meta-analysis, and trial sequential analysis

**DOI:** 10.3389/fmed.2026.1777678

**Published:** 2026-06-23

**Authors:** Yan Pan, Fangjian Chen, Yanzhong Wang

**Affiliations:** 1 Department of Blood Transfusion, The Quzhou Affiliated Hospital of Wenzhou Medical University, Quzhou People’s Hospital, Quzhou, Zhejiang, China; 2 Department of Clinical Laboratory, Sir Run Run Shaw Hospital, Zhejiang University School of Medicine, Hangzhou, Zhejiang, China; 3 School of Medicine, Shaoxing University, Shaoxing, Zhejiang, China

**Keywords:** antigens, cluster of differentiation (CD), immune thrombocytopenia, meta-analysis, polymorphism

## Abstract

**Background:**

Understanding the role of genetic polymorphisms in cluster of differentiation (CD)-related genes is crucial for elucidating susceptibility to immune thrombocytopenia (ITP). This study evaluates the polymorphisms of *CD16*, *CD32*, *CD40*, and *CD152* in ITP patients through a systematic review, meta-analysis, and trial sequential analysis.

**Methods:**

A comprehensive literature review was conducted by a single investigator across multiple databases, including PubMed/Medline, Web of Science, Scopus, and Cochrane Library, up to 7 December 2025. The effect sizes were calculated using Review Manager 5.3 and presented as odds ratios (OR) with a 95% confidence interval. Analyses for publication bias, meta-regression, and sensitivity were performed using Comprehensive Meta-Analysis version 3.0.

**Results:**

Thirty-three articles were included in both qualitative and quantitative syntheses. Significant associations were found for the FcγRIIIA-158 F/V polymorphism across all genetic models, with the highest pooled OR of 2.61 in the homozygous model. No significant associations were observed for the *Fc*γ*RIIA-131 H/R*, *Fc*γ*RIIB-232 I/T*, *CTLA-4 exon 1 A49G*, *CTLA-4 CT60*, *CD40 rs4810485 G* > *T*, and *CD40 rs1883832 C* > *T* polymorphisms. The highest heterogeneity was noted in the heterozygous model for *Fc*γ*RIIA-131 H/R* (I^2^ = 69%), the allelic model for *Fc*γ*RIIB-232 I/T* (I^2^ = 60%), the allelic model for *CTLA-4 exon 1 A49G* (I^2^ = 43%), the dominant model for *CTLA-4 CT60* (I^2^ = 80%), and the allelic model for *CD40 rs1883832 C* > *T* (I^2^ = 87%). The *CD40 rs4810485 G* > *T* polymorphism showed no heterogeneity (I^2^ = 0%) in most models.

**Conclusion:**

The *Fc*γ*RIIIA-158 F/V* polymorphism is consistently associated with disease susceptibility across various genetic models. Other polymorphisms did not show significant associations, indicating the need for further research to validate these findings.

## 1 Introduction

Immune thrombocytopenia (ITP), which was previously known as immune thrombocytopenic purpura (1), is an acquired autoimmune disorder marked by isolated thrombocytopenia due to increased platelet destruction and reduced platelet production (2). Since there is no definitive diagnostic test for ITP, it remains a diagnosis of exclusion, made after ruling out other potential causes of thrombocytopenia (3). ITP is categorized based on its duration into newly diagnosed, persistent (3 to < 12 months), and chronic (≥ 12 months) (3, 4). In adults, ITP typically follows a chronic course (5), while about 80%–90% of children experience spontaneous remission within weeks to months of onset (6).

ITP predominantly affects young adults (7), with the highest incidence occurring between the ages of 15 and 40, and its frequency decreases with advancing age (8). Patients with persistent or chronic ITP face an elevated risk of various comorbidities, including hematologic malignancies (9). Additionally, there is a heightened risk of cardiovascular disease in ITP patients, which is further increased in those who have undergone splenectomy (10). Hemorrhagic symptoms and treatment-related complications, such as infections and cardiovascular diseases, can significantly impact the quality of life of ITP patients (11). Epidemiological studies have also shown an increased incidence of thrombosis in individuals with ITP (11).

Damaging variants in genes related to cellular immunity are more frequently found in children with chronic ITP compared to controls (12). A notable number of ITP patients have a positive family history, suggesting a genetic predisposition to the disease (13). Typically, ITP is caused by immunoglobulin G (IgG) autoantibodies that opsonize the patient’s platelets, leading to significantly increased Fc receptor (FcR)-mediated phagocytosis and destruction by macrophages in the spleen’s reticuloendothelial system (14–17). Considering the involvement of B and T cells in producing autoantibodies against platelet glycoproteins, genetic variations in cluster of differentiation (CD) molecules involved in immune regulation may influence individual susceptibility to ITP (18).

Fc gamma receptors (FcγRs), including FcγRIIa (CD32a), FcγRIIB (CD32b), and FcγRIIIA (CD16), are glycoproteins responsible for binding the Fc fragment of immunoglobulin G (IgG) (19). Intravenous immunoglobulin treatment for ITP works by having exogenous Ig bind to these FcγRs, thereby inhibiting the overactive immune responses in ITP patients (20). FcγRII and FcγRIII, which bind immune complexes with lower affinity than FcγRI, are encoded in the FcγR locus on chromosome 1q23.3, while FcγRI is located on 1q21.2 (21). Cytotoxic T lymphocyte antigen-4 (CTLA-4 or CD152), expressed on T lymphocytes, inhibits T-cell responses (22, 23). The CTLA-4 gene, located on chromosome 2 (2q33), has several identified polymorphisms (24). CD40, a co-stimulatory molecule in the tumor necrosis factor family, consists of 277 amino acids with a molecular weight of 40–50 kDa (25). It is encoded by a gene on chromosome 20q11-13, which includes eight introns and nine exons (26).

Genetic polymorphisms in CD-related immune regulatory genes may contribute to inter-individual differences in susceptibility to immune thrombocytopenia (27). Three meta-analyses have examined FcγR polymorphisms in ITP cases: one (28) analyzed *Fc*γ*RIIIA-158 F/V*, *Fc*γ*RIIA-131 H/R*, and *Fc*γ*RIIB-232 I/T* across 14, 13, and 7 studies, respectively; another (29) focused on *Fc*γ*RIIIA-158 F/V* and *Fc*γ*RIIA-131 H/R*; and a third (30) reviewed *Fc*γ*RIIA-131 H/R*. However, no meta-analyses have comprehensively evaluated the association between *CD40* and *CD152* gene polymorphisms and susceptibility to ITP. Therefore, we conducted a systematic review and meta-analysis to assess the association between polymorphisms in *CD16*, *CD32*, *CD40*, and *CD152* genes and the risk of developing ITP.

## 2 Materials and methods

### 2.1 Study design

The meta-analysis adhered to the guidelines set by the Preferred Reporting Items for Systematic Reviews and Meta-Analyses (PRISMA) (31) (please see PRISMA checklist in Supplementary Material 1). The research question, formulated using the PECO (population, exposure, comparison, and outcome) model, was: Are CD16, CD32, CD40, and CD152 polymorphisms linked to ITP susceptibility in studies involving case and control groups? The study has not been registered in any database.

### 2.2 Identification of articles

A thorough literature review was carried out by a single investigator (YPa) across multiple databases, including PubMed/Medline, Web of Science, Scopus, and Cochrane Library, up to 7 December 2025, without any restrictions to identify relevant studies. The same investigator reviewed the titles and abstracts of the identified articles and obtained the full texts of those that met the inclusion criteria. The search strategy included terms such as: (“immune thrombocytopenic purpura” or “ITP” or “idiopathic thrombocytopenic purpura” or “immune thrombocytopenia” or “idiopathic thrombocytopenia” or “autoimmune thrombocytopenic purpura” or “thrombocytopenia purpura”) and (“FCGR*” or “Fc gamma receptor” or “Fcγ receptor*” or “FcγR*” or “CD32*” or “CD16*” or “FCgammaR*” or “CTLA-4” OR “Cytotoxic T lymphocyte antigen-4” or “cytotoxic T-lymphocyte antigen 4” or “CTLA4” or “CD152” or “CD40”) and (“variant*” or “mutation*” or “genotype*” or “allele*” or “polymorphism*”). To ensure no relevant study was missed, the reference lists of the articles were also examined. The search and selection process was verified by another author (YW). In case of any disagreement between the two authors, a third author resolved it.

### 2.3 Eligibility criteria

The inclusion criteria for our analysis were as follows:

(a) Studies must have designed case and control groups examining the *CD16*, *CD32*, *CD40*, and *CD152* polymorphisms in individuals diagnosed with ITP and in control subjects.

(b) The diagnosis of ITP in patients could be based on clinical and laboratory findings.

(c) ITP patients included in the studies should not have any other systemic diseases, and control subjects should be either healthy individuals or those without any hematological disorders.

Conversely, we excluded the following types of articles from our analysis:

(a) Review articles, meta-analyses, and systematic reviews.

(b) Articles with incomplete data or those without a control group.

(c) Animal studies.

(d) Conference papers and commentaries.

(e) Duplicate studies and book chapters.

(f) Studies where the control group included individuals with the disease.

### 2.4 Data summary

Two authors (LH and FC) independently gathered data from the studies included in the meta-analysis. Any discrepancies were resolved through mutual discussion.

### 2.5 Quality evaluation

The quality assessment of the studies was performed by one author using the Newcastle-Ottawa Scale (NOS) tool (32), a well-established instrument for evaluating the quality of non-randomized studies in meta-analyses. The maximum possible score a study can achieve is nine (33). Two authors (YC and YPe) independently evaluated the scores of each study. Any disagreements between them were resolved through discussion, ensuring a thorough and consensus-based evaluation process.

### 2.6 Statistical analyses

The effect sizes were computed using the Review Manager 5.3 (RevMan 5.3) software and presented as odds ratios (OR) with a 95% confidence interval (CI), representing the prevalence of alleles/genotypes of *CD16*, *CD32*, *CD40*, and *CD152* polymorphisms in patients with ITP and control subjects. The significance of the pooled OR was determined using the Z-test, with a two-sided *p*-value of less than 0.05 considered significant. A random-effects model (34) was employed if *P*_*heterogeneity*_ was < 0.10 (I^2^ > 50%), indicating significant heterogeneity. If heterogeneity was not significant, a fixed-effect model (35) was applied.

Subgroup analyses were performed to determine if there were significant differences in the pooled ORs within these groups. Additionally, a meta-regression analysis using a random-effects model was conducted to demonstrate a linear relationship between the auxiliary variables in the study and the effect size. Begg’s funnel plot and Begg’s test were used to evaluate the likelihood of publication bias, and the degree of asymmetry was assessed with Egger’s test. The *p*-values from both Egger’s and Begg’s tests were calculated, with a two-sided *p*-value less than 0.10 indicating the presence of publication bias.

Sensitivity analysis was conducted using two methods to assess the stability and consistency of the pooled ORs: (1) The “one-study-removed” analysis and (2) The “cumulative” analysis. All analyses related to publication bias, meta-regression, and sensitivity analysis were performed using the Comprehensive Meta-Analysis version 3.0 (CMA 3.0) software.

To reduce the risk of drawing false-positive or negative conclusions from meta-analyses (36), a trial sequential analysis (TSA) was performed using the TSA software (version 0.9.5.10 beta) (37). The TSA allows for the establishment of a futility threshold, which can determine a non-effect result before reaching the necessary information size. The required information size (RIS) was calculated with an alpha risk of 5%, a beta risk of 20%, and a two-sided boundary type. If the Z-curve reached the RIS line or traced the boundary line or futility area, it suggested that the studies included a sufficient number of cases and that the conclusions were reliable. If not, it indicated that the information available was insufficient and additional data was required. The O’Brien–Fleming alpha-spending function was applied to construct trial sequential monitoring boundaries. The cumulative Z-curve was plotted against the monitoring boundaries to determine whether the available evidence was sufficient to confirm or refute an association. TSA was performed only for polymorphisms with an adequate number of studies (generally ≥ 8–10 studies) to ensure reliable sequential monitoring. Therefore, TSA was conducted for *Fc*γ*RIIIA-158 F/V*, *Fc*γ*RIIA-131 H/R*, and *Fc*γ*RIIB-232 I/T (695T* > *C)* polymorphisms, while *CTLA-4* and *CD40* polymorphisms were not analyzed due to the limited number of available studies.

## 3 Results

### 3.1 Study selection

**FIGURE 1:**
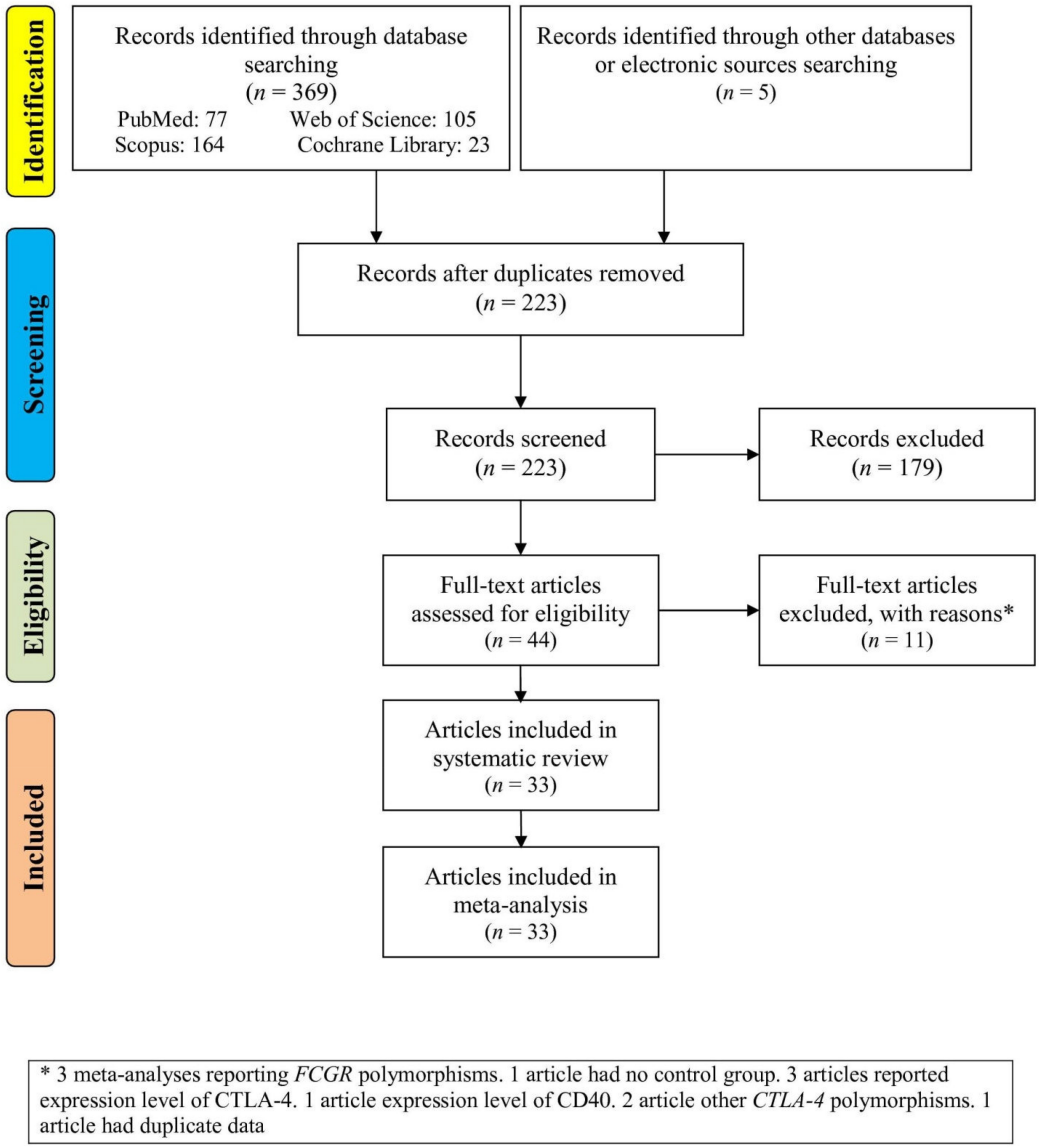
Flowchart of the study selection.

The flowchart in Figure 1 illustrates the study selection process for a systematic review or meta-analysis. Initially, 369 records were identified through database searches, including PubMed, Web of Science, Cochrane Library, and Scopus, and 5 records along with other electronic sources. After removing duplicates, 223 records remained for screening. Of these, 179 were excluded, leaving 44 full-text articles assessed for eligibility. Ultimately, 33 articles (38–70) were included in the qualitative synthesis, and then in the quantitative synthesis (meta-analysis).

### 3.2 Studies’ characteristics

Table 1 shows the characteristics of the study included in the meta-analysis reporting FcγR polymorphisms (CD16 and CD32) and Table 2 for CD40 and CTLA-4 (CD152) polymorphisms. The studies were published from 1998 to 2024.

**TABLE 1:** Characteristics of the study included in the meta-analysis reporting *Fc*γ*R* polymorphisms (*CD16* and *CD32*).

References	Ethnicity	Disease type	Sample size (cases/controls)	Allele frequency		Genotyping distribution		*p*-value for HWE	NOS score
				Cases	Controls	Cases	Controls		
***Fc*γ*RIIIA-158 F/V***				**F/V**		**FF/FV/VV**			
Amorim et al. (41)	Mixed	Childhood-onset ITP	32/73	30/34	97/49	10/10/12	36/25/12	**0.047**	9
Audia et al. (42)	Caucasian	Adult-onset ITP	23/109	23/23	151/67	NA	NA	NA	8
Breunis et al. (43)	Caucasian	Adult-onset ITP	44/98	55/33	138/58	19/17/8	48/42/8	0.778	9
Breunis et al. (43)	Caucasian	Childhood-onset ITP	72/98	72/72	138/58	16/40/16	48/42/8	0.778	9
Bruin et al. (44)	Caucasian	Childhood-onset ITP	53/154	51/55	205/103	12/27/14	66/73/15	0.421	9
Carcao et al. (45)	Mixed	Childhood-onset ITP	97/129	112/82	178/80	30/52/15	62/54/13	0.806	8
Eyada et al. (48)	Caucasian	Childhood-onset ITP	92/89	106/78	130/48	24/58/10	47/36/6	0.800	9
Foster et al. (49)	Mixed	Childhood-onset ITP	37/191	49/25	262/120	13/23/1	92/78/21	0.470	7
Fujimoto et al. (50)	Asian	Adult-onset ITP	104/59	121/87	80/38	28/65/11	25/30/4	0.206	9
Mohamed et al. (55)	Caucasian	Childhood-onset ITP	55/55	52/58	42/68	11/30/14	14/14/27	**< 0.001**	8
Nourse et al. (56)	Mixed	Adult-onset ITP	100/100	106/94	140/60	27/52/21	48/44/8	0.634	9
Papagianni et al. (57)	Caucasian	Childhood-onset ITP	53/45	58/48	59/31	6/46/1	15/29/1	**0.004**	8
Pavkovic et al. (59)	Caucasian	Adult-onset ITP	125/120	132/118	150/90	40/52/33	52/46/22	**0.046**	9
Schmidt et al. (63)	Caucasian	Childhood-onset ITP	180/180	182/124	236/124	62/58/33	79/78/23	0.587	8
Wang et al. (64)	Caucasian	Adult-onset ITP	74/111	67/81	126/96	11/45/18	34/58/19	0.497	9
Zakaria et al. (69)	Caucasian	Childhood-onset ITP	80/80	74/86	112/48	8/58/14	40/32/8	0.670	8
Zhu et al. (70)	Caucasian	Adult-onset ITP	44/97	56/32	141/53	21/14/9	49/43/5	0.252	9
***Fc*γ*RIIA-131 H/R***				**H/R**		**HH/HR/RR**			
Amorim et al. (41)	Mixed	Childhood-onset ITP	33/73	38/28	80/66	10/18/5	20/40/13	0.365	9
Audia et al. (42)	Caucasian	Adult-onset ITP	24/108	33/15	118/98	NA	NA	NA	8
Breunis et al. (43)	Caucasian	Adult-onset ITP	44/100	36/42	108/92	10/26/8	28/52/20	0.641	9
Breunis et al. (43)	Caucasian	Childhood-onset ITP	72/100	78/66	108/92	25/28/19	28/52/20	0.641	9
Bruin et al. (44)	Caucasian	Childhood-onset ITP	52/154	40/44	162/146	12/26/14	40/82/32	0.400	9
Carcao et al. (45)	Mixed	Childhood-onset ITP	96/130	77/115	131/129	18/41/37	31/69/30	0.482	8
Eyada et al. (48)	Caucasian	Childhood-onset ITP	92/90	149/35	162/18	65/19/8	72/18/0	0.292	9
Foster et al. (49)	Mixed	Childhood-onset ITP	36/218	55/37	231/205	16/13/7	67/97/54	0.114	7
Fujimoto et al. (50)	Asian	Adult-onset ITP	104/59	137/71	74/44	40/57/7	22/30/7	0.503	9
Horsewood et al. (52)	Mixed	Childhood-onset ITP	28/100	34/22	104/96	10/14/4	26/52/22	0.677	9
Mohamed et al. (55)	Caucasian	Childhood-onset ITP	55/55	62/48	71/49	18/26/11	14/33/8	0.112	8
Pavkovic et al. (59)	Caucasian	Adult-onset ITP	125/120	158/92	160/80	50/58/17	55/50/15	0.494	9
Rajantie et al. (61)	Caucasian	Adult-onset ITP	15/6	16/14	10/2	4/8/3	4/2/0	0.624	7
Schmidt et al. (63)	Caucasian	Childhood-onset ITP	180/180	187/173	197/163	46/95/39	55/87/38	0.741	8
Williams et al. (65)	Caucasian	Adult-onset ITP	29/51	50/18	55/47	14/12/3	15/25/11	0.923	9
Zakaria et al. (69)	Caucasian	Childhood-onset ITP	80/80	82/78	120/40	18/46/16	56/8/16	**< 0.001**	8
Zhu et al. (70)	Caucasian	Adult-onset ITP	44/94	54/34	119/69	18/18/8	40/39/15	0.299	9
***Fc*γ*RIIB-232 I/T (695T* > *C)***				**I/T**		**II/IT/TT**			
Audia et al. (42)	Caucasian	Adult-onset ITP	24/108	44/4	190/26	NA	NA	NA	8
Breunis et al. (43)	Caucasian	Childhood-onset ITP	44/100	79/9	177/23	37/5/2	81/15/4	**0.009**	9
Breunis et al. (43)	Caucasian	Adult-onset ITP	72/100	130/14	177/23	60/10/2	81/15/4	**0.009**	9
Bruin et al. (44)	Caucasian	Childhood-onset ITP	55/148	104/6	265/31	49/6/0	118/29/1	0.585	9
He et al. (51)	Asian	Childhood-onset ITP	76/37	116/36	67/7	42/32/2	30/7/0	0.525	8
Satoh et al. (62)	Asian	Adult-onset ITP	206/193	310/112	324/62	113/84/9	134/56/3	0.291	8
Schmidt et al. (63)	Caucasian	Childhood-onset ITP	180/180	314/46	317/43	137/40/3	143/31/6	**0.015**	8
Xu et al. (66)	Asian	Childhood-onset ITP	102/243	153/51	353/133	54/45/3	130/93/20	0.561	9
Xu et al. (66)	Asian	Adult-onset ITP	178/243	255/101	353/133	93/69/16	130/93/20	0.561	9
Zhu et al. (70)	Caucasian	Adult-onset ITP	44/97	70/18	147/47	26/18/0	50/47/0	**0.002**	9

The bold number is statistically significant (*p* < 0.05). NOS, Newcastle-Ottawa Scale; ITP, immune thrombocytopenia; *Fc*γ*R*, Fc gamma receptors; CD, cluster of differentiation; HWE, Hardy-Weinberg equilibrium.

**TABLE 2:** Characteristics of the study included in the meta-analysis reporting *CD40* and *CTLA-4 (CD152)* polymorphisms.

References	Ethnicity	Disease type	Sample size (cases/controls)	Allele frequency		Genotyping distribution		*p*-value for HWE	NOS score
				Cases	Controls	Cases	Controls		
*CTLA-4 exon 1 A49G (rs231775)*				A/G		AA/AG/GG			
Aktürk et al. (40)	Caucasian	Adult-onset ITP	36/150	59/13	215/85	23/13/0	72/71/7	**0.042**	9
El Demerdash et al. (46)	Caucasian	Adult-onset ITP	88/44	53/123	34/54	9/35/44	5/24/15	0.318	9
Kasamatsu et al. (53)	Asian	Adult-onset ITP	119/219	94/144	173/265	19/56/44	31/111/77	0.371	7
Pavkovic et al. (58)	Caucasian	Onset ITP	60/100	79/41	141/59	26/27/7	51/39/10	0.532	6
Radwan and Goda (60)	Caucasian	Childhood-onset ITP	100/259	139/61	391/127	47/45/8	142/107/10	0.061	8
***CTLA-4 CT60 (rs3087243)***				**A/G**		**AA/AG/GG**			
El Demerdash et al. (46)	Caucasian	Adult-onset ITP	88/44	99/77	70/18	34/31/23	35/0/9	**< 0.001**	9
Kasamatsu et al. (53)	Asian	Adult-onset ITP	119/219	62/176	125/313	9/44/66	16/93/110	0.542	7
Li et al. (54)	Asian	Childhood-onset ITP	186/162	57/315	52/272	4/49/133	3/46/113	0.494	8
Yao et al. (68)	Asian	Childhood-onset ITP	102/175	58/146	88/262	7/44/51	8/72/95	0.218	9
***CD40 rs4810485 G* > *T***				**G/T**		**GG/GT/TT**			
Abd El Dayem et al. (38)	Caucasian	Childhood-onset ITP	60/60	82/38	84/36	31/20/9	29/26/5	0.805	8
AbdelGhafar et al. (39)	Caucasian	Adult-onset ITP	101/97	89/113	89/105	26/37/38	24/41/32	0.142	9
Ellithy et al. (47)	Caucasian	Adult-onset ITP	50/50	32/68	24/76	4/24/22	4/16/30	0.385	8
Yan et al. (67)	Asian	Adult-onset ITP	160/160	107/213	94/226	16/75/69	10/74/76	0.146	9
***CD40 rs1883832 C* > *T***				**C/T**		**CC/CT/TT**			
Abd El Dayem et al. (38)	Caucasian	Childhood-onset ITP	60/60	82/38	84/36	31/20/9	29/26/5	0.805	8
AbdelGhafar et al. (39)	Caucasian	Adult-onset ITP	101/97	53/149	71/123	7/39/55	15/41/41	0.379	9
Ellithy et al. (47)	Caucasian	Adult-onset ITP	50/50	32/68	32/68	4/24/22	2/28/20	**0.042**	7
Yan et al. (67)	Asian	Adult-onset ITP	160/160	211/109	152/168	77/57/26	39/74/47	0.357	9

The bold number is statistically significant (*p* < 0.05). NOS, Newcastle-Ottawa Scale; ITP, immune thrombocytopenia; *Fc*γ*R*, Fc gamma receptors; CD, cluster of differentiation; CTLA-4, cytotoxic T lymphocyte antigen-4; HWE, Hardy-Weinberg equilibrium.

### 3.3 Pooled analysis

A summary of pooled analyses for all polymorphisms is shown in Table 3. The forest plots are located in Supplementary Material 2. For the FcγRIIIA-158 F/V polymorphism, significant associations were found in all genetic models, with the highest pooled OR observed in the homozygous model (OR = 2.61, 95% CI: 2.05–3.33, *p* < 0.00001, I^2^ = 38%). The FcγRIIA-131 H/R polymorphism showed no significant associations in any model, with the highest heterogeneity (I^2^ = 69%) in the heterozygous model. The FcγRIIB-232 I/T (695T > C) polymorphism also showed no significant associations, with the highest heterogeneity (I^2^ = 60%) in the allelic model. For the CTLA-4 exon 1 A49G (rs231775) polymorphism, no significant associations were found, with the highest heterogeneity (I^2^ = 43%) in the allelic model. The CTLA-4 CT60 (rs3087243) polymorphism showed no significant associations, with the highest heterogeneity (I^2^ = 80%) in the dominant model. The CD40 rs4810485 G > T polymorphism showed no significant associations, with no heterogeneity (I^2^ = 0%) in most models. Lastly, the CD40 rs1883832 C > T polymorphism showed no significant associations, with the highest heterogeneity (I^2^ = 87%) in the allelic model.

**TABLE 3:** Summary of pooled analyses for all polymorphisms.

Polymorphism	Genetic model	Number of studies	OR	95% CI		*p*-value	I^2^
				Min.	Max.		
*Fc*γ*RIIIA-158 F/V*	Allelic	17	1.63	1.38	1.92	**< 0.00001**	55%
	Homozygous	16	2.61	2.05	3.33	**< 0.00001**	38%
	Heterozygous	16	1.99	1.51	2.63	**< 0.00001**	58%
	Dominant	16	2.12	1.66	2.71	**< 0.00001**	52%
	Recessive	16	1.74	1.40	2.15	**< 0.00001**	50%
*Fc*γ*RIIA-131 H/R*	Allelic	17	1.09	0.90	1.32	0.37	60%
	Homozygous	16	1.22	0.96	1.55	0.10	32%
	Heterozygous	16	1.12	0.79	1.60	0.52	69%
	Dominant	16	1.15	0.83	1.58	0.41	68%
	Recessive	16	1.16	0.94	1.43	0.16	8%
*Fc*γ*RIIB-232 I/T (695T* > *C)*	Allelic	10	1.06	0.81	1.40	0.67	60%
	Homozygous	9	0.99	0.64	1.53	0.95	11%
	Heterozygous	9	1.20	0.99	1.46	0.06	46%
	Dominant	9	1.12	0.83	1.51	0.44	53%
	Recessive	9	0.92	0.60	1.43	0.72	0%
*CTLA-4 exon 1 A49G (rs231775)*	Allelic	5	1.11	0.92	1.34	0.28	43%
	Homozygous	5	1.20	0.77	1.89	0.42	4%
	Heterozygous	5	1.01	0.75	1.36	0.96	6%
	Dominant	5	1.06	0.80	1.41	0.68	28%
	Recessive	5	1.29	0.92	1.81	0.14	0%
*CTLA-4 CT60 (rs3087243)*	Allelic	4	1.26	0.81	1.95	0.30	76%
	Homozygous	4	1.25	0.76	2.06	0.38	35%
	Heterozygous	4	1.49	0.36	6.15	0.58	76%
	Dominant	4	1.41	0.46	4.36	0.55	80%
	Recessive	4	1.08	0.84	1.39	0.56	0%
*CD40 rs4810485 G* > *T*	Allelic	4	0.91	0.73	1.13	0.39	0%
	Homozygous	4	0.92	0.57	1.47	0.72	0%
	Heterozygous	4	0.78	0.51	1.19	0.25	0%
	Dominant	4	0.83	0.56	1.24	0.37	0%
	Recessive	4	0.92	0.68	1.26	0.62	35%
*CD40 rs1883832 C* > *T*	Allelic	4	0.94	0.50	1.77	0.84	87%
	Homozygous	4	0.92	0.25	3.40	0.90	84%
	Heterozygous	4	0.70	0.33	1.50	0.36	66%
	Dominant	4	0.77	0.31	1.96	0.59	80%
	Recessive	4	1.08	0.53	2.21	0.83	75%

The bold number is statistically significant (*p* < 0.05). *Fc*γ*R*, Fc gamma receptors; CD, cluster of differentiation; CTLA-4, cytotoxic T lymphocyte antigen-4; OR, odds ratio; CI, confidence interval.

### 3.4 Subgroup analysis

Table 4 presents subgroup analyses for three polymorphisms, focusing on different genetic models, ethnicities, and disease types. For the FcγRIIIA-158 F/V polymorphism, significant associations were found in Caucasians and mixed populations across various genetic models, with the highest odds ratios observed in the homozygous model for both adult-onset and childhood-onset ITP. The FcγRIIA-131 H/R polymorphism showed significant associations in the homozygous model for Caucasians and childhood-onset ITP, but not in other subgroups. The FcγRIIB-232 I/T (695T > C) polymorphism did not show significant associations in any subgroup. These results highlight the importance of considering subgroup analyses to understand the variability in genetic associations across different populations and disease types.

**TABLE 4:** Subgroup analyses for three polymorphisms.

Polymorphism	Genetic model	Subgroup	Variable (*N*)	OR	95% CI		*p*-value	I^2^
					Min.	Max.		
*Fc*γ*RIIIA-158 F/V*	Allelic	Ethnicity	Caucasian (12)	1.62	1.30	2.02	**< 0.0001**	64%
			Asian (1)	1.51	0.94	2.43	0.09	–
			Mixed (4)	1.71	1.36	2.15	**< 0.00001**	29%
		Disease type	Adult-onset ITP (7)	1.64	1.38	1.96	**< 0.00001**	0%
			Childhood-onset ITP (10)	1.61	1.23	2.12	**0.0006**	72%
	Homozygous	Ethnicity	Caucasian (11)	2.61	1.97	3.47	**< 0.00001**	46%
			Asian (1)	2.46	0.69	8.70	0.16	–
			Mixed (4)	2.65	1.62	4.34	**0.0001**	46%
		Disease type	Adult-onset ITP (6)	2.79	1.89	4.11	**< 0.00001**	0%
			Childhood-onset ITP (10)	2.70	1.59	4.58	**0.0002**	58%
	Heterozygous	Ethnicity	Caucasian (11)	2.04	1.40	2.98	**0.0002**	69%
			Asian (1)	1.93	0.97	3.86	0.06	–
			Mixed (4)	1.97	1.40	2.78	0.0001	0%
		Disease type	Adult-onset ITP (6)	1.55	1.17	2.04	**0.002**	26%
			Childhood-onset ITP (10)	2.41	1.61	3.59	**< 0.0001**	65%
	Dominant	Ethnicity	Caucasian (11)	2.19	1.52	3.14	**< 0.0001**	67%
			Asian (1)	2.00	1.02	3.92	**0.04**	–
			Mixed (4)	2.12	1.53	2.94	**< 0.00001**	0%
		Disease type	Adult-onset ITP (6)	1.78	1.37	2.31	**< 0.0001**	0%
			Childhood-onset ITP (10)	2.44	1.69	3.53	**< 0.00001**	64%
	Recessive	Ethnicity	Caucasian (11)	1.71	1.33	2.19	**< 0.0001**	57%
			Asian (1)	1.63	0.49	5.36	0.42	–
					**Min.**	**Max.**		
			Mixed (4)	1.89	0.88	4.04	0.10	54%
		Disease type	Adult-onset ITP (6)	2.04	1.45	2.89	**< 0.0001**	0%
			Childhood-onset ITP (10)	1.59	0.96	2.65	0.07	64%
*Fc*γ*RIIA-131 H/R*	Allelic	Ethnicity	Caucasian (12)	1.12	0.87	1.45	0.38	67%
			Asian (1)	0.87	0.54	1.40	0.57	–
			Mixed (4)	1.15	0.90	1.46	0.27	44%
		Disease type	Adult-onset ITP (7)	0.95	0.78	1.17	0.65	43%
			Childhood-onset ITP (10)	1.20	0.93	1.54	0.15	66%
	Homozygous	Ethnicity	Caucasian (11)	1.37	1.03	1.82	**0.03**	22%
			Asian (1)	0.55	0.17	1.77	0.32	–
			Mixed (4)	0.88	0.40	1.92	0.74	56%
		Disease type	Adult-onset ITP (6)	0.96	0.61	1.50	0.85	9%
			Childhood-onset ITP (10)	1.34	1.01	1.78	**0.04**	42%
	Heterozygous	Ethnicity	Caucasian (11)	1.31	0.80	2.14	0.29	77%
			Asian (1)	1.04	0.53	2.07	0.90	–
			Mixed (4)	0.79	0.53	1.19	0.26	0%
		Disease type	Adult-onset ITP (6)	1.12	0.82	1.54	0.49	0%
			Childhood-onset ITP (10)	1.13	0.66	1.93	0.65	80%
	Dominant	Ethnicity	Caucasian (11)	1.28	0.86	1.91	0.23	72%
			Asian (1)	0.95	0.49	1.84	0.88	–
			Mixed (4)	0.85	0.58	1.25	0.41	20%
		Disease type	Adult-onset ITP (6)	1.08	0.80	1.46	0.60	24%
			Childhood-onset ITP (10)	1.17	0.74	1.87	0.50	77%
	Recessive	Ethnicity	Caucasian (11)	1.19	0.92	1.52	0.18	0%
			Asian (1)	0.54	0.18	1.61	0.27	–
			Mixed (4)	1.21	0.81	1.81	0.34	54%
		Disease type	Adult-onset ITP (6)	0.89	0.59	1.36	0.60	0%
			Childhood-onset ITP (10)	1.27	1.00	1.61	0.05	19%
***Fc*γ*RIIB-232 I/T (695T* > *C)***	Allelic	Ethnicity	Caucasian (6)	0.85	0.65	1.12	0.25	0%
			Asian (4)	1.40	0.89	2.20	0.15	80%
		Disease type	Adult-onset ITP (6)	1.09	0.73	1.62	0.69	67%
			Childhood-onset ITP (5)	1.03	0.68	1.56	0.91	56%
	Homozygous	Ethnicity	Caucasian (5)	0.69	0.29	1.69	0.42	0%
			Asian (4)	1.22	0.46	3.22	0.69	54%
			Adult-onset ITP (5)	1.36	0.77	2.40	0.29	32%
			Childhood-onset ITP (5)	0.60	0.29	1.26	0.18	0%
	Heterozygous	Disease type	Caucasian (5)	0.91	0.66	1.27	0.59	5%
			Asian (4)	1.45	0.98	2.14	0.06	57%
		Ethnicity	Adult-onset ITP (5)	1.20	0.93	1.56	0.16	50%
			Childhood-onset ITP (5)	1.17	0.72	1.90	0.52	54%
	Dominant	Disease type	Caucasian (5)	0.88	0.64	1.21	0.43	0%
			Asian (4)	1.46	0.94	2.26	0.09	68%
		Ethnicity	Adult-onset ITP (5)	1.22	0.95	1.56	0.11	59%
			Childhood-onset ITP (5)	1.11	0.69	1.79	0.67	57%
	Recessive		Caucasian (5)	0.69	0.29	1.68	0.42	0%
			Asian (4)	1.02	0.62	1.68	0.94	49%
		Disease type	Adult-onset ITP (5)	1.28	0.73	2.25	0.38	7%
			Childhood-onset ITP (5)	0.56	0.27	1.16	0.12	0%

*N*, number of studies. The bold number is statistically significant (*p* < 0.05). *Fc*γ*R*, Fc gamma receptors; OR, odds ratio; CI, confidence interval.

### 3.5 Sensitivity analysis

Both “one-study-removed” and “cumulative” analyses did not change the pooled results and therefore, the pooled results were stable. Removing four studies (41, 55, 57, 59) with a deviation of Hardy-Weinberg equilibrium (HWE) in their control groups for FcγRIIIA-158 F/V polymorphism, reduced the publication bias in allelic and recessive models to 0%. For FcγRIIA-131 H/R polymorphism, removing one study (69), the publication bias was reduced in allelic, heterozygous, and dominant models significantly. For FcγRIIB-232 I/T (695T > C), removing four studies from three articles (43, 63, 70), the results did not change significantly.

### 3.6 Meta-regression analysis

Table 5 presents meta-regression analyses for three polymorphisms, examining the impact of publication year, sample size, and *p*-value of HWE on the pooled ORs across different genetic models. For the FcγRIIIA-158 F/V polymorphism, significant associations were found with the publication year in the allelic (coefficient = 0.0003, *p* = 0.0458), homozygous (coefficient = 0.0006, *p* = 0.0306), heterozygous (coefficient = 0.0006, *p* = 0.0089), and dominant (coefficient = 0.0006, *p* = 0.0039) models. Additionally, the *p*-value of HWE showed a significant association in the allelic model (coefficient = 0.6340, *p* = 0.0204). For the FcγRIIA-131 H/R polymorphism, the *p*-value of HWE was significantly associated with the allelic model (coefficient = −0.9411, *p* = 0.0088). No significant associations were found for the FcγRIIB-232 I/T (695T > C) polymorphism in any model. These results suggest that publication year and HWE can influence the observed associations in meta-analyses of genetic polymorphisms.

**TABLE 5:** Meta-regression analyses for three polymorphisms.

Polymorphism	Genetic model	Variable	Coefficient	95% CI		*Z*-value	*p*-value
				Min.	Max.		
*Fc*γ*RIIIA-158 F/V*	Allelic	Publication year	0.0003	< 0.00001	0.0005	2.00	**0.0458**
		Sample size	−0.0020	−0.0045	0.0005	−1.54	0.1239
		*p*-value of HWE	0.6340	0.0981	1.1700	2.32	**0.0204**
	Homozygous	Publication year	0.0006	0.0001	0.0011	2.16	**0.0306**
		Sample size	−0.0030	−0.0079	0.0020	−1.18	0.2391
		*p*-value of HWE	1.0389	−0.0518	2.1296	1.87	0.0619
	Heterozygous	Publication year	0.0006	0.0001	0.0010	2.62	**0.0089**
		Sample size	−0.0039	−0.0085	0.0006	−1.71	0.0857
		*p*-value of HWE	0.5315	−0.4186	1.4815	1.10	0.2729
	Dominant	Publication year	0.0006	0.0002	0.0009	2.88	**0.0039**
		Sample size	−0.0035	−0.0073	0.0003	−1.79	0.0742
		*p*-value of HWE	0.6277	−0.1858	1.4412	1.51	0.1305
	Recessive	Publication year	0.0002	−0.0004	0.0007	0.55	0.5820
		Sample size	−0.0003	−0.0060	0.0055	−0.09	0.9309
		*p*-value of HWE	0.7357	−0.4670	1.9385	1.20	0.2306
*Fc*γ*RIIA-131 H/R*	Allelic	Publication year	0.0002	−0.0001	0.0004	1.19	0.2346
		Sample size	0.0013	−0.0010	0.0035	1.10	0.2699
		*p*-value of HWE	−0.9411	−1.6455	−0.2368	−2.62	**0.0088**
	Homozygous	Publication year	0.0001	−0.0003	0.0006	0.52	0.6019
		Sample size	0.0017	−0.0024	0.0059	0.81	0.4167
		*p*-value of HWE	−0.9857	−2.3134	0.3420	−1.46	0.1457
	Heterozygous	Publication year	0.0003	−0.0003	0.0009	1.02	0.3084
		Sample size	0.0003	−0.0046	0.0052	0.12	0.9057
		*p*-value of HWE	−1.1657	−2.6752	0.3438	−1.51	0.1301
	Dominant	Publication year	0.0003	−0.0002	0.0007	0.99	0.3208
		Sample size	0.0006	−0.0037	0.0049	0.27	0.7841
		*p*-value of HWE	−1.0649	−2.3802	0.2503	−1.59	0.1152
	Recessive	Publication year	< 0.0001	−0.0003	0.0004	0.22	0.8291
		Sample size	0.0009	−0.0023	0.0042	0.56	0.5758
		*p*-value of HWE	−0.3449	−1.4200	0.7303	−0.63	0.5296
*Fc*γ*RIIB-232 I/T (695T* > *C)*	Allelic	Publication year	−0.0001	−0.0005	0.0004	−0.25	0.8018
		Sample size	0.005	−0.0024	0.0035	0.36	0.7222
		*p*-value of HWE	0.1182	−1.2268	1.4631	0.17	0.8633
	Homozygous	Publication year	−0.0001	−0.0011	0.0010	−0.14	0.8884
		Sample size	0.0003	−0.0067	0.0073	0.09	0.9290
		*p*-value of HWE	0.0748	−2.9618	3.1114	0.05	0.9615
	Heterozygous	Publication year	−0.0001	−0.0005	0.0004	−0.30	0.7644
		Sample size	0.0008	−0.0022	0.0037	0.52	0.6057
		*p*-value of HWE	0.1450	−1.2071	1.4972	0.21	0.8335
	Dominant	Publication year	−0.0001	−0.0005	0.0004	−0.27	0.7847
		Sample size	0.0006	−0.0024	0.0037	0.40	0.6919
		*p*-value of HWE	0.1866	−1.2105	1.5837	0.26	0.7935
	Recessive	Publication year	−0.0001	−0.0011	0.0009	−0.20	0.8428
		Sample size	0.0004	−0.0063	0.0070	0.11	0.9121
		*p*-value of HWE	−0.0064	−2.8660	2.8533	< −0.01	0.9965

The bold number is statistically significant (*p* < 0.05). *Fc*γ*R*, Fc gamma receptors; CI, confidence interval; HWE, Hardy-Weinberg equilibrium.

### 3.7 Publication bias

Table 6 represents the results of publication bias tests for various polymorphisms using Egger’s and Begg’s tests. For the *Fc*γ*RIIIA-158 F/V* polymorphism, the heterozygous (*p* = 0.0885) and dominant (*p* = 0.0644) models showed potential publication bias according to Egger’s test, though these values are just below the significance threshold (*p* < 0.10). For the *Fc*γ*RIIA-131 H/R* polymorphism, none of the genetic models showed significant publication bias in either test. Similarly, for the *Fc*γ*RIIB-232 I/T (695T* > *C)* polymorphism, no significant publication bias was detected. The *CTLA-4 exon 1 A49G (rs231775)* and *CTLA-4 CT60 (rs3087243)* polymorphisms also did not show significant publication bias in any model. For the *CD40 rs4810485 G* > *T* and *CD40 rs1883832 C* > *T* polymorphisms, no significant publication bias was observed. These results suggest that, overall; there is minimal evidence of publication bias for the polymorphism of *Fc*γ*RIIIA-158 F/V*. Supplementary Material 3 shows the funnel plots for the polymorphisms.

**TABLE 6:** Results of the tests of publication bias for all polymorphisms.

Polymorphism	Genetic model	Number of studies	*p*-value of Egger’s test	*p*-value of Begg’s test
*Fc*γ*RIIIA-158 F/V*	Allelic	17	0.3409	0.6803
	Homozygous	16	0.7987	0.4713
	Heterozygous	16	**0.0885**	0.2074
	Dominant	16	**0.0644**	0.2799
	Recessive	16	0.7834	0.8571
*Fc*γ*RIIA-131 H/R*	Allelic	17	0.7834	0.1613
	Homozygous	16	0.9361	0.1496
	Heterozygous	16	0.7754	0.5284
	Dominant	16	0.8586	0.3678
	Recessive	16	0.8604	0.2074
*Fc*γ*RIIB-232 I/T (695T* > *C)*	Allelic	10	0.4252	0.4208
	Homozygous	9	0.9593	0.6206
	Heterozygous	9	0.4099	0.5316
	Dominant	9	0.4773	0.2971
	Recessive	9	0.9779	0.6206
*CTLA-4 exon 1 A49G (rs231775)*	Allelic	5	0.6270	0.6242
	Homozygous	5	0.7584	0.6242
	Heterozygous	5	0.3492	0.3271
	Dominant	5	0.4662	0.3271
	Recessive	5	0.9185	0.6242
*CTLA-4 CT60 (rs3087243)*	Allelic	4	0.1272	0.4969
	Homozygous	4	0.5753	0.4969
	Heterozygous	4	0.1596	0.1742
	Dominant	4	0.5687	0.4969
	Recessive	4	0.6788	0.4969
*CD40 rs4810485 G* > *T*	Allelic	4	0.8953	0.4969
	Homozygous	4	0.9068	1.000
	Heterozygous	4	0.2816	1.000
	Dominant	4	0.9506	0.4969
	Recessive	4	0.7185	0.4969
*CD40 rs1883832 C* > *T*	Allelic	4	0.3306	1.000
	Homozygous	4	0.4260	1.000
	Heterozygous	4	0.5092	0.4969
	Dominant	4	0.4515	0.4969
	Recessive	4	0.5417	0.4969

The bold number is statistically significant (*p* < 0.10). *Fc*γ*R*, Fc gamma receptors; CD, cluster of differentiation; CTLA-4, cytotoxic T lymphocyte antigen-4.

### 3.8 TSA

Three polymorphisms of *Fc*γ*R* were analyzed for TSA that had sufficient studies. Supplementary Material 4 shows the TSA plots for the polymorphisms. The results showed that the Z-curve crossed the RIS line for *Fc*γ*RIIIA-158 F/V* polymorphism. TSA demonstrated that the cumulative Z-curve for the *Fc*γ*RIIIA-158 F/V* polymorphism crossed both the conventional significance boundary and the RIS boundary, indicating that the available evidence is sufficient and the association is unlikely to be a false-positive finding. In contrast, for the *Fc*γ*RIIA-131 H/R* and *Fc*γ*RIIB-232 I/T (695T* > *C)* polymorphisms, the cumulative Z-curves did not cross the RIS or trial sequential monitoring boundaries, suggesting that the current evidence remains inconclusive and that additional studies are required to confirm or refute these associations.

## 4 Discussion

The analyses revealed significant associations for the *Fc*γ*RIIIA-158 F/V* polymorphism across all genetic models, with the highest OR in the homozygous model. No significant associations were found for the *Fc*γ*RIIA-131 H/R*, *Fc*γ*RIIB-232 I/T (695T* > *C)*, *CTLA-4 exon 1 A49G (rs231775)*, *CTLA-4 CT60 (rs3087243)*, *CD40 rs4810485 G* > *T*, and *CD40 rs1883832 C* > *T* polymorphisms. Publication bias tests indicated potential bias in some models for the *Fc*γ*RIIIA-158 F/V* polymorphism, but not for the other polymorphisms. TSA confirmed the reliability of the pooled results for the *Fc*γ*RIIIA-158 F/V* polymorphism while suggesting the need for more studies for the *Fc*γ*RIIA-131 H/R* and *Fc*γ*RIIB-232 I/T (695T* > *C)* polymorphisms. These findings indicate that the evidence supporting the association between the *Fc*γ*RIIIA-158 F/V* polymorphism and ITP susceptibility is robust, whereas the absence of significant associations for other polymorphisms may reflect insufficient statistical power rather than a definitive lack of association.

The absence of significant associations for several polymorphisms, including *Fc*γ*RIIA-131 H/R*, *Fc*γ*RIIB-232 I/T (695T* > *C)*, *CTLA-4 exon 1 A49G (rs231775)*, *CTLA-4 CT60 (rs3087243)*, and *CD40 rs4810485 G* > *T*, and *rs1883832 C* > *T*, should be interpreted with caution. In some cases, these findings may indicate a true lack of association with susceptibility to immune thrombocytopenia. However, for polymorphisms with a limited number of available studies, particularly *CTLA-4* and *CD40* variants, the statistical power of the meta-analysis may be insufficient to detect modest genetic effects. Additionally, the presence of moderate to high heterogeneity in certain models and the results of TSA suggesting that the required information size has not been reached further support the need for additional well-designed studies. Therefore, the current evidence does not definitively exclude a potential association between these loci and ITP susceptibility, and future large-scale studies in diverse populations are warranted.

A substantial amount of evidence indicates that the development of ITP may be linked to a critical genetic alteration that causes immunologic system dysfunction and thrombocytopenia (19, 71–73). Numerous well-known pathologies could account for thrombocytopenia, including B and T cell responses, cytokine balance disruptions, anti-platelet antibody production, phagocytic cell activation, surface molecule changes, and immune cell dysfunctions with variations in the Th1/Th2 ratio (74, 75). Given the direct association of certain CD markers with chronic ITP, genetic variations in CD-related immune regulatory genes may influence immune responses and susceptibility to chronic ITP (18). Additionally, the interaction between genetic factors and environmental triggers plays a crucial role in the pathogenesis of ITP. Environmental factors, such as infections or medications, can trigger the onset or worsening of ITP in genetically predisposed individuals (76, 77).

The etiology of ITP is highly complex. To better understand the potential roles of genetic variations in ITP, we strongly recommend that future studies conduct haplotype analyses and investigate potential gene-gene interactions (28). These interactions can reveal how different genetic variants collaborate to influence immune regulation and platelet production (76, 78). Genetic variants are believed to play a significant role in susceptibility, particularly in persistent or chronic ITP (79). Several *CD40* gene variants have been associated with autoimmune and inflammatory diseases such as Graves’ disease and rheumatoid arthritis (80). CTLA-4, also known as CD152, acts as a competitive antagonist for B7 on the surface of antigen-presenting cells and is responsible for T-cell inactivation and immune tolerance due to its involvement in immune checkpoint pathways (81). Polymorphisms in *CD152*-encoding genes are implicated in the pathogenesis of many autoimmune diseases (82). Furthermore, understanding the role of immune checkpoint pathways, such as those involving CD152, in ITP could open new avenues for therapeutic interventions. Immune checkpoints are crucial regulators of immune responses, and their dysregulation can lead to autoimmune conditions. Although the link between immune checkpoints and ITP is not fully established, exploring this connection could provide valuable insights into disease mechanisms and potential treatment targets (79, 83).

Phagocytosis of autoantibody-sensitized platelets via FcRs on phagocytic cells is a key mechanism of thrombocytopenia in primary ITP (84). Several studies have indicated that genetic alterations in FcγRIIa and/or FcγRIIIa can modify the receptor-binding affinity with immunoglobulins (Igs) (48, 85, 86). Polymorphisms in FcRs have been linked to various diseases, including heparin-induced thrombocytopenia (86) and systemic lupus erythematosus (SLE) (45), and have been associated with clinical responses to rituximab-based treatments in patients with follicular lymphoma (87). Understanding these genetic variations is essential for developing personalized therapeutic strategies and improving clinical outcomes in patients with immune-mediated conditions (88).

In summary, ITP development is a complex interplay of genetic and immunological factors leading to thrombocytopenia. Genetic changes can disrupt immune system functions, resulting in various pathologies such as altered B and T cell responses, cytokine imbalances, and the production of anti-platelet antibodies. Evaluation of genetic variations in CD-related genes may contribute to understanding susceptibility and immune dysregulation in ITP. Additionally, genetic predispositions combined with environmental triggers play a significant role in the disease’s onset and progression. Future research should delve into haplotype analyses and gene-gene interactions to uncover the genetic underpinnings of ITP. Investigating immune checkpoint pathways, like those involving CD152, could reveal new therapeutic targets. Understanding genetic variations, especially in *FcRs*, is crucial for developing personalized treatments and enhancing clinical outcomes in immune-mediated conditions.

Emerging evidence suggests that genetic susceptibility may contribute not only to the development of ITP but also to variability in treatment response and the risk of refractory disease in both pediatric and adult populations. Refractory ITP, defined as persistent thrombocytopenia despite standard therapies, represents a major clinical challenge and is associated with increased morbidity and healthcare burden (89, 90).

Polymorphisms in immune regulatory genes, particularly those encoding FcγRs, may influence treatment outcomes by altering antibody-mediated platelet clearance and immune complex handling. For example, functional variants in *Fc*γ*RIIA* and *Fc*γ*RIIIA* can modify receptor affinity for IgG, thereby affecting macrophage-mediated phagocytosis of opsonized platelets and potentially influencing responsiveness to therapies such as intravenous immunoglobulin (IVIG) or corticosteroids. Similarly, polymorphisms in inhibitory receptors such as *Fc*γ*RIIB* may disrupt immune tolerance mechanisms and contribute to persistent immune activation and treatment resistance (91).

In addition to *Fc*γ*R* genes, polymorphisms in co-stimulatory and immune checkpoint molecules, including *CTLA-4* and *CD40*, may play a role in refractoriness by regulating T-cell activation, B-cell differentiation, and autoantibody production (79). Dysregulation of these pathways may lead to sustained immune-mediated platelet destruction and reduced responsiveness to immunosuppressive therapies. These mechanisms are particularly relevant in chronic or refractory ITP (89), where immune dysregulation persists despite standard treatment (83).

Furthermore, genetic variability may contribute to differences in disease phenotype between pediatric and adult patients. Pediatric ITP is often acute and self-limited, whereas adult ITP is more frequently chronic and treatment-resistant (91).

Genetic factors influencing immune regulation, inflammatory signaling, and platelet destruction pathways may partially explain these differences and may serve as potential biomarkers for predicting treatment response and disease prognosis.

Overall, the integration of genetic susceptibility data with clinical outcomes may improve risk stratification and guide personalized therapeutic strategies in ITP. Future studies investigating the association between specific genetic polymorphisms and treatment response are warranted to better understand the mechanisms underlying refractoriness and to support precision medicine approaches in the management of ITP.

### 4.1 Limitations

Common limitations of studies like the one summarized include (1) Sample size: Small sample sizes in most polymorphisms could limit the statistical power of the study, making it difficult to detect significant associations or generalize findings to larger populations. Small number of available studies for some polymorphisms, particularly *CTLA-4* and *CD40* variants may reduce statistical power and increase the risk of false-negative findings. (2) Heterogeneity: High heterogeneity (I^2^ values) among some polymorphisms (particularly *CTLA-4 CT60* and *CD40 rs1883832*) could indicate variability in study outcomes, which may be due to differences in study design, population characteristics, or other factors, complicating the interpretation of pooled results. (3) Publication bias: The presence of publication bias in one polymorphism, where studies with positive results are more likely to be published, could skew the overall findings. (4) Genetic and environmental interactions: The study may not account for interactions between genetic polymorphisms and environmental factors, which can influence the observed associations. (5) HWE: Deviations from HWE in the included studies can indicate potential issues with the study population or genotyping errors, affecting the reliability of the results. (6) Study design variability: Differences in study design, such as case-control versus cohort studies, can introduce bias and affect the comparability of results across studies. (7) Ethnic diversity: Limited ethnic diversity in the study populations can restrict the applicability of the findings to other ethnic groups. (8) Functional studies: The lack of functional studies to elucidate the biological mechanisms underlying the genetic associations can limit the understanding of how these polymorphisms contribute to disease. (9) Registration: Lack of registration in a prospective database (e.g., PROSPERO) may introduce potential bias.

## 5 Conclusion

The *Fc*γ*RIIIA-158 F/V* polymorphism shows consistent and significant associations across various genetic models, indicating its potential role in disease susceptibility. In contrast, other polymorphisms analyzed did not show significant associations, highlighting the need for further research to confirm these findings. The significant associations found for the *Fc*γ*RIIIA-158 F/V* polymorphism suggest that it could be a valuable genetic marker for predicting susceptibility to certain diseases, particularly those involving immune system dysfunctions. Identifying individuals with this polymorphism could help in early diagnosis and personalized treatment strategies. Future studies should focus on increasing the sample size and diversity of populations studied to confirm the associations found for the *Fc*γ*RIIIA-158 F/V* polymorphism and to explore potential associations for other polymorphisms. Additionally, integrating functional studies to understand the biological mechanisms underlying these associations will be crucial for translating these findings into clinical practice.

## Data Availability

The datasets presented in this study can be found in online repositories. The names of the repository/repositories and accession number(s) can be found in this article/Supplementary material.
