# Supplementary material for: Evaluation of CD16, CD32, CD40, and CD152 polymorphisms in immune thrombocytopenia patients: a systematic review, meta-analysis, and trial sequential analysis
Source: Front Med (Lausanne). 2026 Jun 23;13:1777678. doi: 10.3389/fmed.2026.1777678 (PMC13337454; doi:10.3389/fmed.2026.1777678)
Supplement: Supplementary file 1 [file Supplementary_file_1.docx]

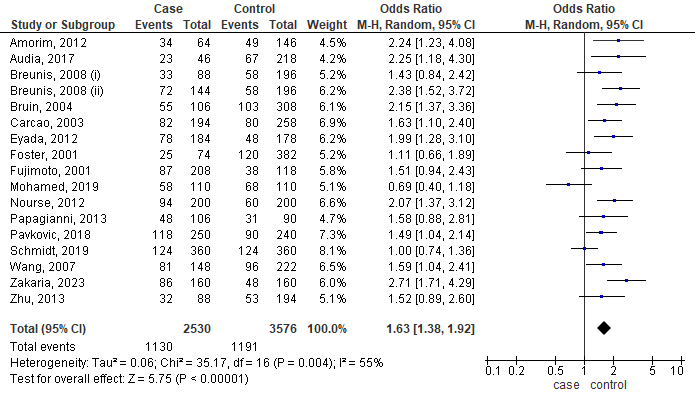


**Figure 1**: Forest plot of association of *FcγRIIIA-158 F/V* polymorphism with idiopathic thrombocytopenic purpura susceptibility in allelic model


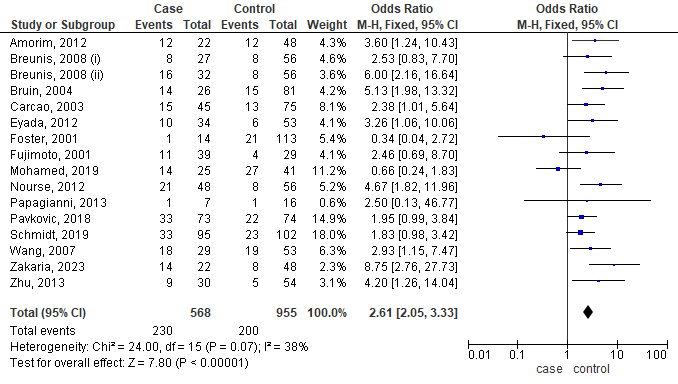


**Figure 2**: Forest plot of association of *FcγRIIIA-158 F/V* polymorphism with idiopathic thrombocytopenic purpura susceptibility in homozygous model


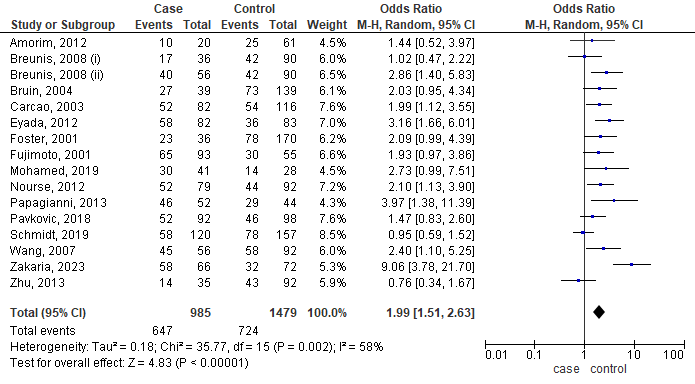


**Figure 3**: Forest plot of association of *FcγRIIIA-158 F/V* polymorphism with idiopathic thrombocytopenic purpura susceptibility in heterozygous model


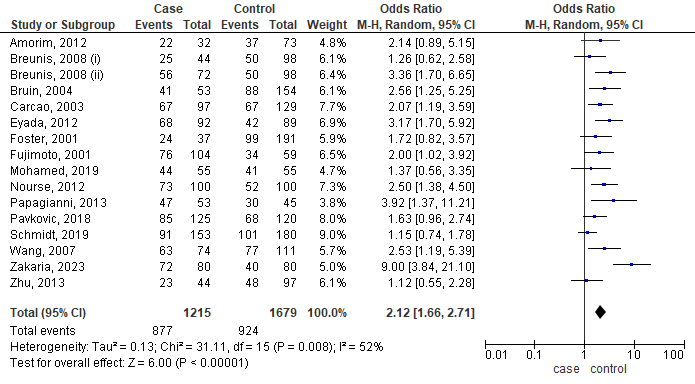


**Figure 4**: Forest plot of association of *FcγRIIIA-158 F/V* polymorphism with idiopathic thrombocytopenic purpura susceptibility in dominant model


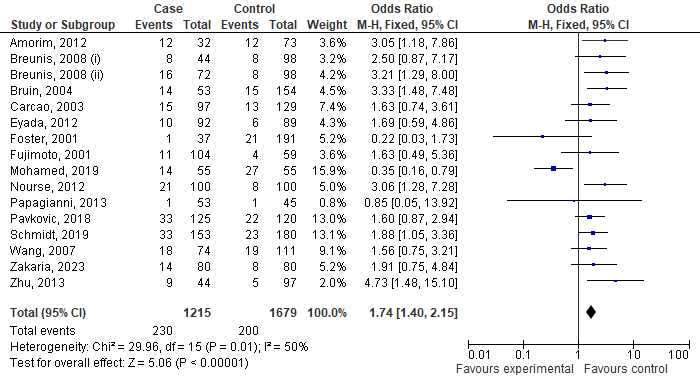


**Figure 5**: Forest plot of association of *FcγRIIIA-158 F/V* polymorphism with idiopathic thrombocytopenic purpura susceptibility in recessive model


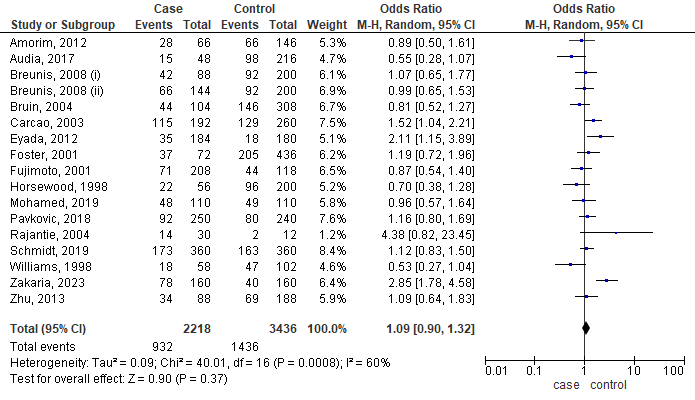


**Figure 6**: Forest plot of association of *FcγRIIA-131 H/R* polymorphism with idiopathic thrombocytopenic purpura susceptibility in allelic model


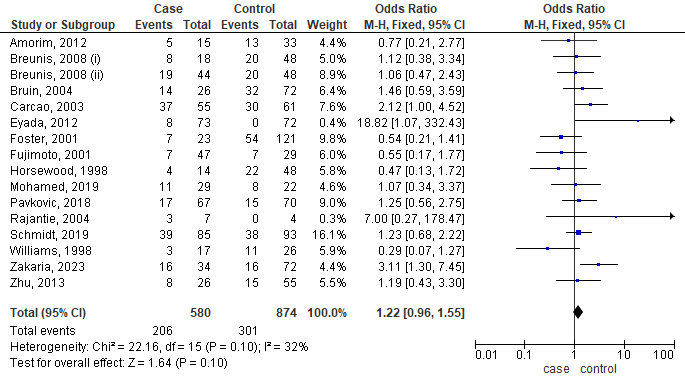


**Figure 7**: Forest plot of association of *FcγRIIA-131 H/R* polymorphism with idiopathic thrombocytopenic purpura susceptibility in homozygous model


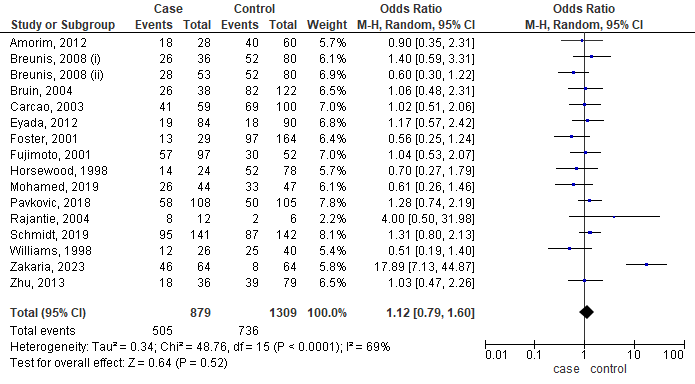


**Figure 8**: Forest plot of association of *FcγRIIA-131 H/R* polymorphism with idiopathic thrombocytopenic purpura susceptibility in heterozygous model


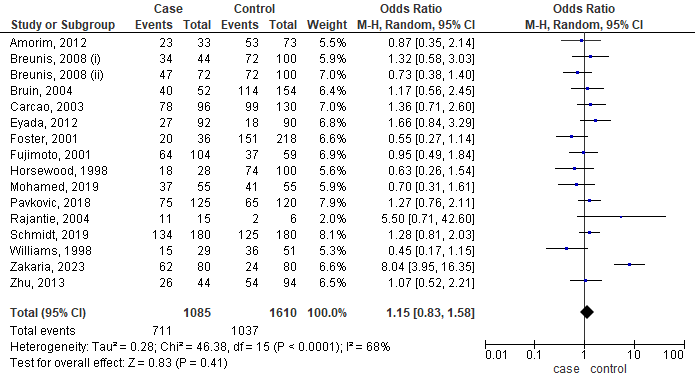


**Figure 9**: Forest plot of association of *FcγRIIA-131 H/R* polymorphism with idiopathic thrombocytopenic purpura susceptibility in dominant model


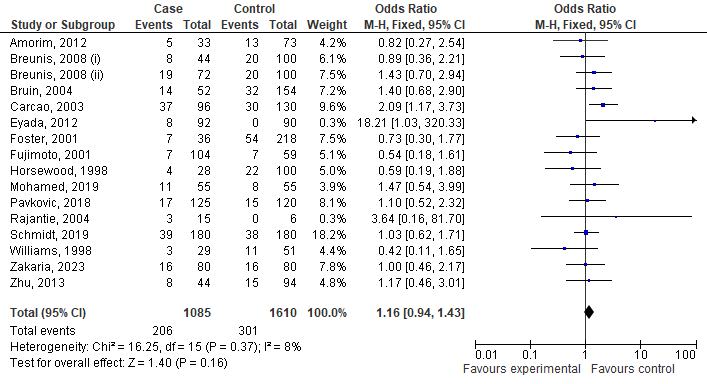


**Figure 10**: Forest plot of association of *FcγRIIA-131 H/R* polymorphism with idiopathic thrombocytopenic purpura susceptibility in recessive model


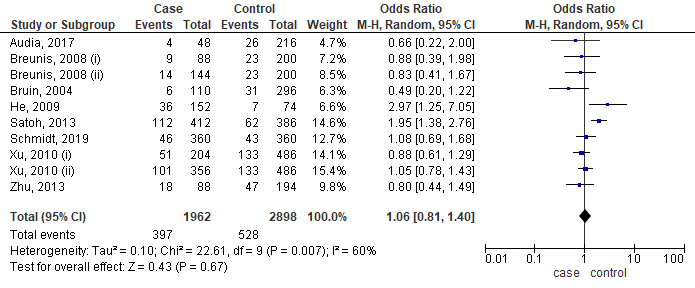


**Figure 11**: Forest plot of association of *FcγRIIB-232 I/T* polymorphism with idiopathic thrombocytopenic purpura susceptibility in allelic model


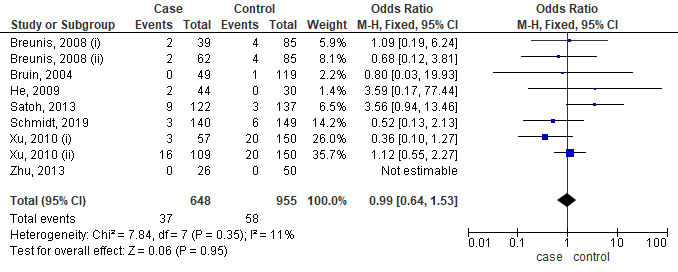


**Figure 12**: Forest plot of association of *FcγRIIB-232 I/T* polymorphism with idiopathic thrombocytopenic purpura susceptibility in homozygous model


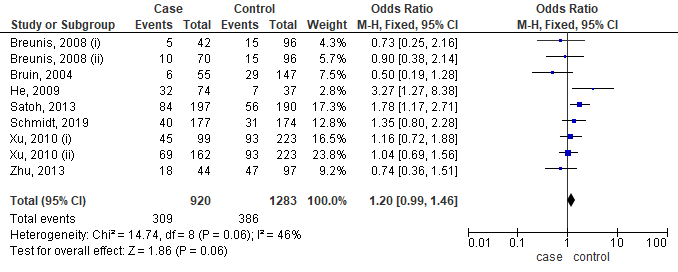


**Figure 13**: Forest plot of association of *FcγRIIB-232 I/T* polymorphism with idiopathic thrombocytopenic purpura susceptibility in heterozygous model


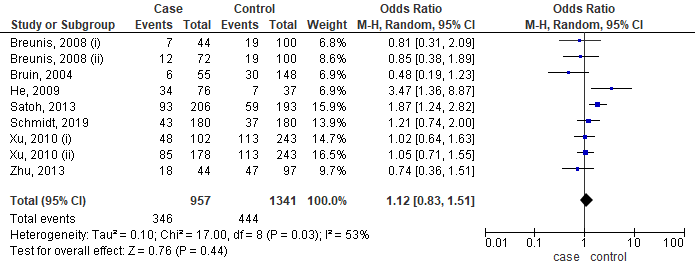


**Figure 14**: Forest plot of association of *FcγRIIB-232 I/T* polymorphism with idiopathic thrombocytopenic purpura susceptibility in dominant model


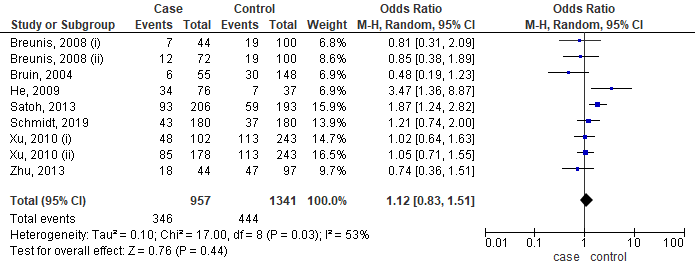


**Figure 15**: Forest plot of association of *FcγRIIB-232 I/T* polymorphism with idiopathic thrombocytopenic purpura susceptibility in recessive model


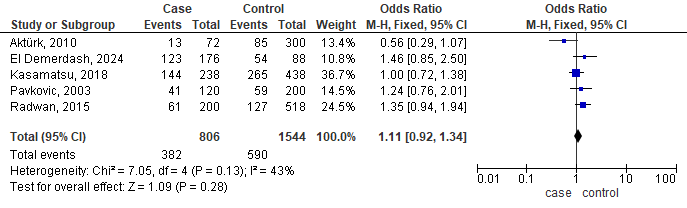


**Figure 16**: Forest plot of association of *CTLA-4 exon 1 A49G* polymorphism with idiopathic thrombocytopenic purpura susceptibility in allelic model


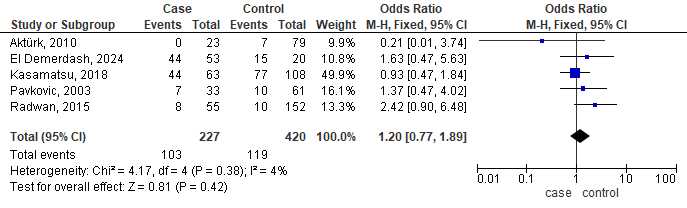


**Figure 17**: Forest plot of association of *CTLA-4 exon 1 A49G* polymorphism with idiopathic thrombocytopenic purpura susceptibility in homozygous model


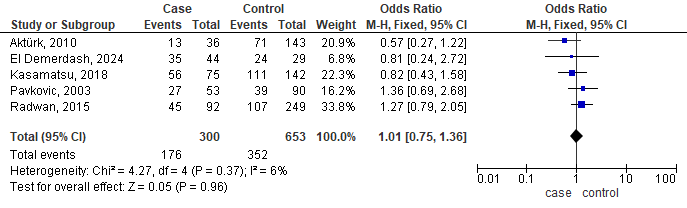


**Figure 18**: Forest plot of association of *CTLA-4 exon 1 A49G* polymorphism with idiopathic thrombocytopenic purpura susceptibility in heterozygous model


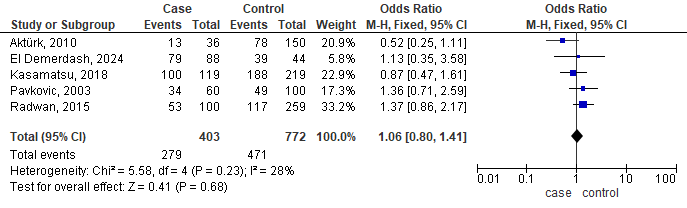


**Figure 19**: Forest plot of association of *CTLA-4 exon 1 A49G* polymorphism with idiopathic thrombocytopenic purpura susceptibility in dominant model


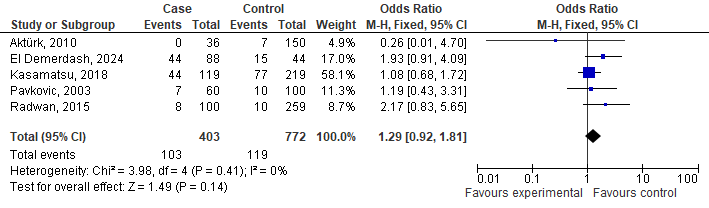


**Figure 20**: Forest plot of association of *CTLA-4 exon 1 A49G* polymorphism with idiopathic thrombocytopenic purpura susceptibility in recessive model


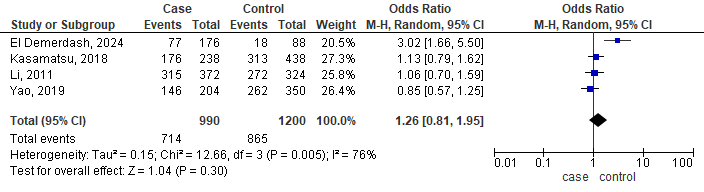


**Figure 21**: Forest plot of association of *CTLA-4 CT60* polymorphism with idiopathic thrombocytopenic purpura susceptibility in allelic model


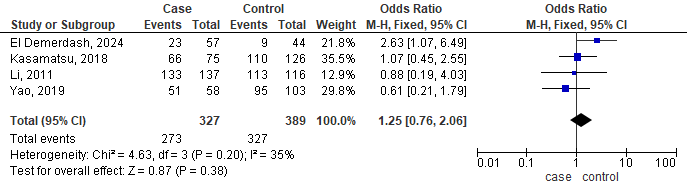


**Figure 22**: Forest plot of association of *CTLA-4 CT60* polymorphism with idiopathic thrombocytopenic purpura susceptibility in homozygous model


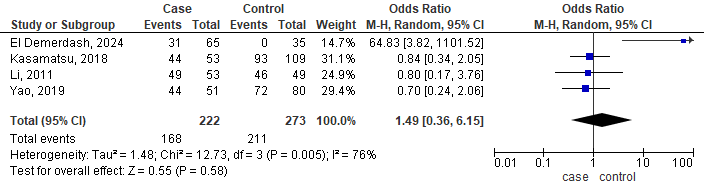


**Figure 23**: Forest plot of association of *CTLA-4 CT60* polymorphism with idiopathic thrombocytopenic purpura susceptibility in heterozygous model


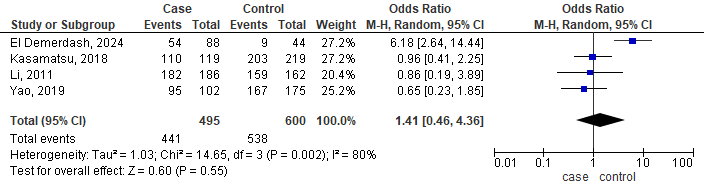


**Figure 24**: Forest plot of association of *CTLA-4 CT60* polymorphism with idiopathic thrombocytopenic purpura susceptibility in dominant model


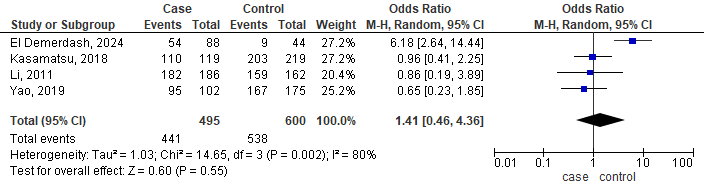


**Figure 25**: Forest plot of association of *CTLA-4 CT60* polymorphism with idiopathic thrombocytopenic purpura susceptibility in recessive model

**
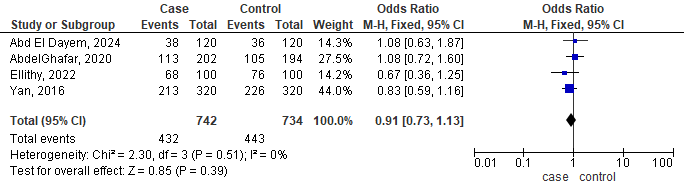
**

**Figure 26**: Forest plot of association of *CD40 rs4810485* polymorphism with idiopathic thrombocytopenic purpura susceptibility in allelic model


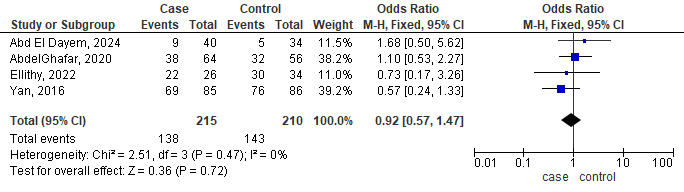


**Figure 27**: Forest plot of association of *CD40 rs4810485* polymorphism with idiopathic thrombocytopenic purpura susceptibility in homozygous model


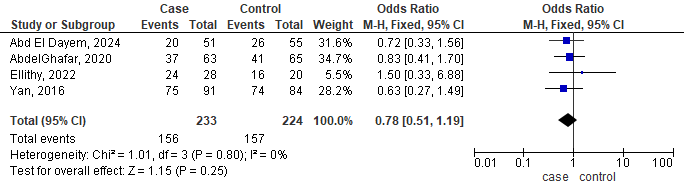


**Figure 28**: Forest plot of association of *CD40 rs4810485* polymorphism with idiopathic thrombocytopenic purpura susceptibility in heterozygous model


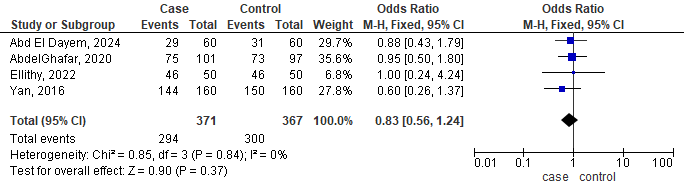


**Figure 29**: Forest plot of association of *CD40 rs4810485* polymorphism with idiopathic thrombocytopenic purpura susceptibility in dominant model


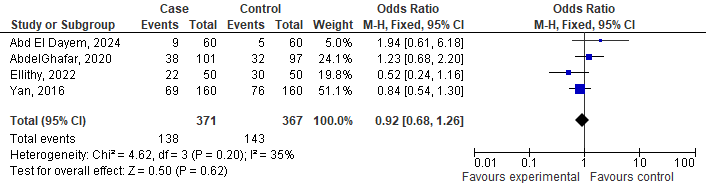


**Figure 30**: Forest plot of association of *CD40 rs4810485* polymorphism with idiopathic thrombocytopenic purpura susceptibility in recessive model

**
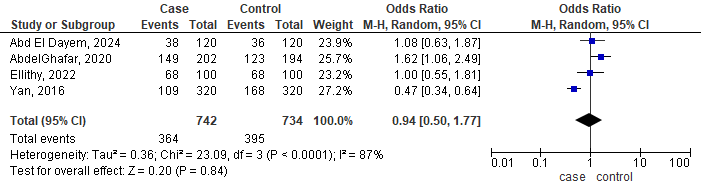
**

**Figure 31**: Forest plot of association of *CD40 rs1883832* polymorphism with idiopathic thrombocytopenic purpura susceptibility in allelic model


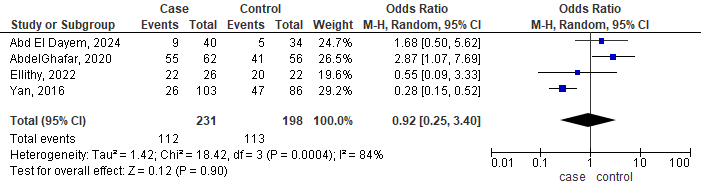


**Figure 32**: Forest plot of association of *CD40 rs1883832* polymorphism with idiopathic thrombocytopenic purpura susceptibility in homozygous model


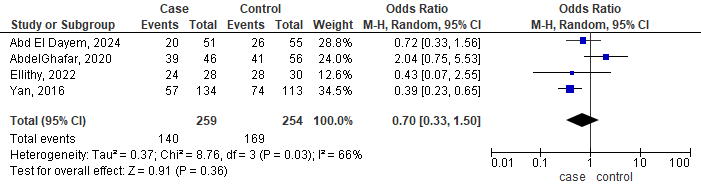


**Figure 33**: Forest plot of association of *CD40 rs1883832* polymorphism with idiopathic thrombocytopenic purpura susceptibility in heterozygous model


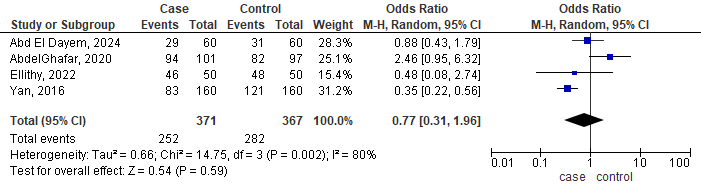


**Figure 34**: Forest plot of association of *CD40 rs1883832* polymorphism with idiopathic thrombocytopenic purpura susceptibility in dominant model


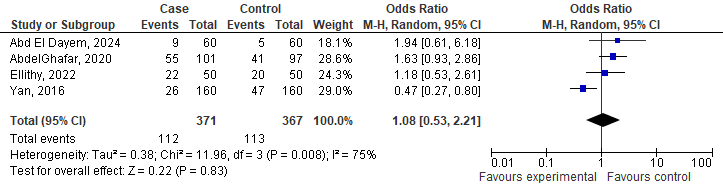


**Figure 35**: Forest plot of association of *CD40 rs1883832* polymorphism with idiopathic thrombocytopenic purpura susceptibility in recessive model
