# Supplementary material for: Evaluation of CD16, CD32, CD40, and CD152 polymorphisms in immune thrombocytopenia patients: a systematic review, meta-analysis, and trial sequential analysis
Source: Front Med (Lausanne). 2026 Jun 23;13:1777678. doi: 10.3389/fmed.2026.1777678 (PMC13337454; doi:10.3389/fmed.2026.1777678)
Supplement: Supplementary file 2 [file Supplementary_file_2.docx]

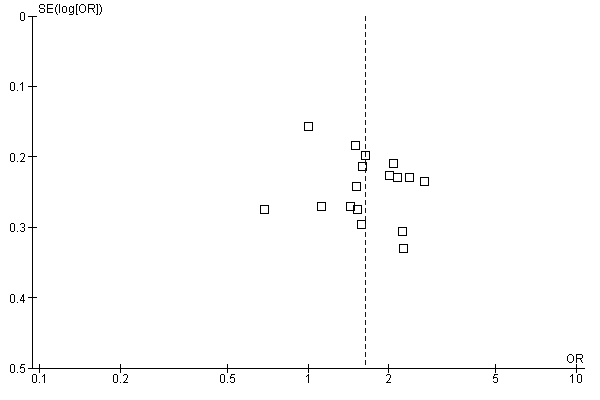


**Figure 1**: Funnel plot of association of *FcγRIIIA-158 F/V* polymorphism with idiopathic thrombocytopenic purpura susceptibility in allelic model


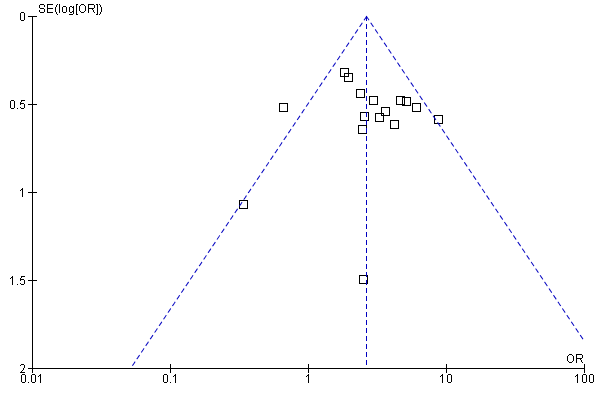


**Figure 2**: Funnel plot of association of *FcγRIIIA-158 F/V* polymorphism with idiopathic thrombocytopenic purpura susceptibility in homozygous model


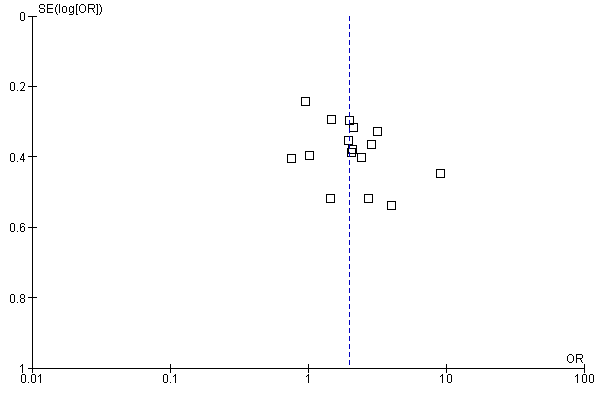


**Figure 3**: Funnel plot of association of *FcγRIIIA-158 F/V* polymorphism with idiopathic thrombocytopenic purpura susceptibility in heterozygous model


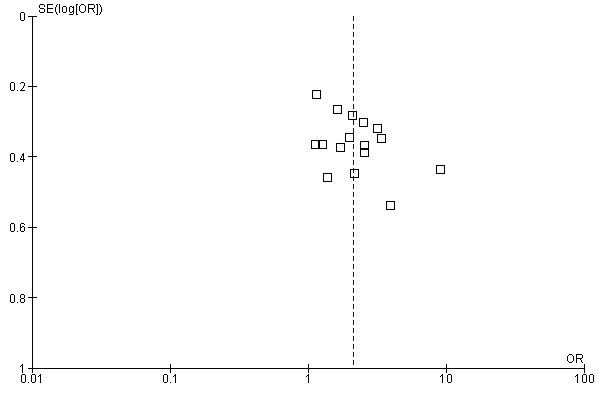


**Figure 4**: Funnel plot of association of *FcγRIIIA-158 F/V* polymorphism with idiopathic thrombocytopenic purpura susceptibility in dominant model


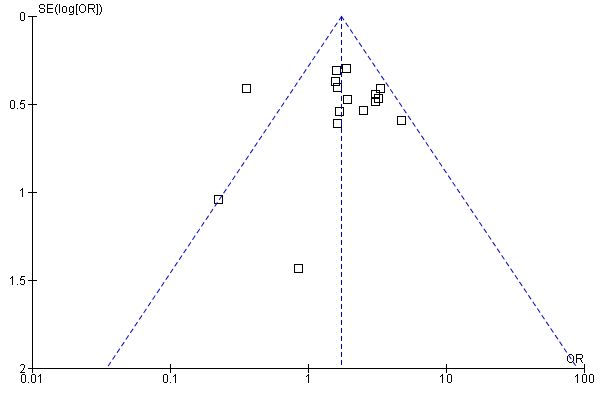


**Figure 5**: Funnel plot of association of *FcγRIIIA-158 F/V* polymorphism with idiopathic thrombocytopenic purpura susceptibility in recessive model


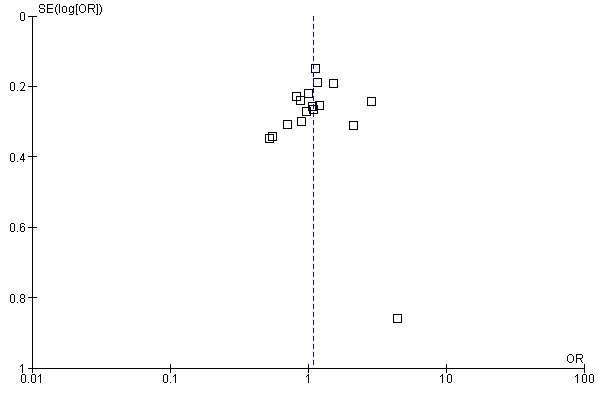


**Figure 6**: Funnel plot of association of *FcγRIIA-131 H/R* polymorphism with idiopathic thrombocytopenic purpura susceptibility in allelic model


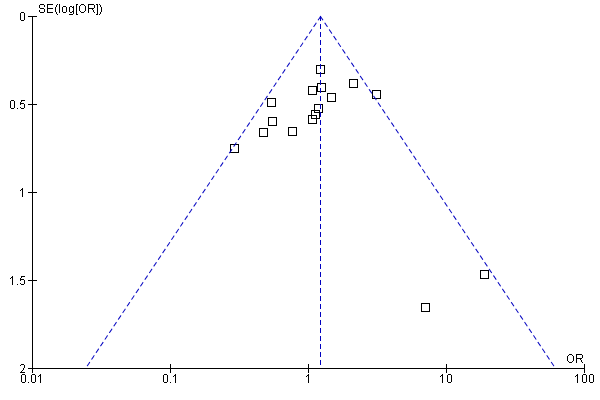


**Figure 7**: Funnel plot of association of *FcγRIIA-131 H/R* polymorphism with idiopathic thrombocytopenic purpura susceptibility in homozygous model


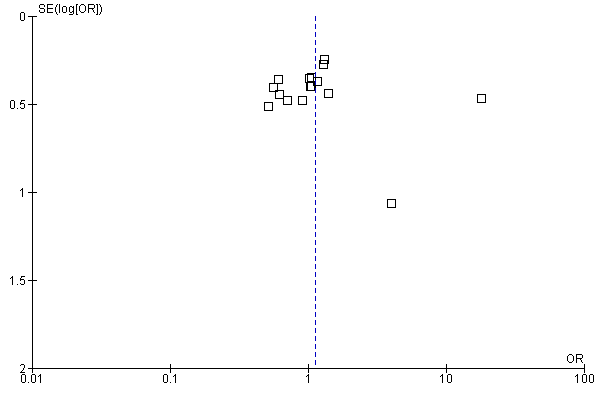


**Figure 8**: Funnel plot of association of *FcγRIIA-131 H/R* polymorphism with idiopathic thrombocytopenic purpura susceptibility in heterozygous model


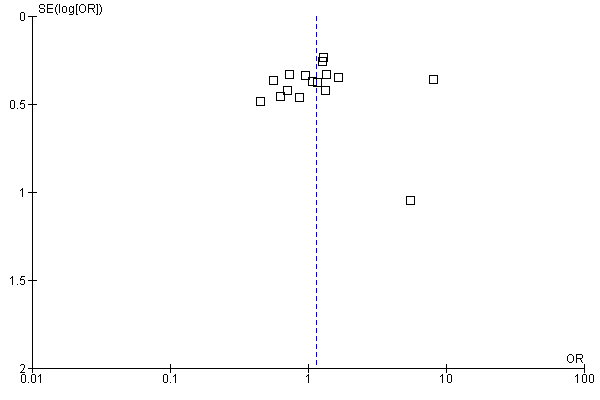


**Figure 9**: Funnel plot of association of *FcγRIIA-131 H/R* polymorphism with idiopathic thrombocytopenic purpura susceptibility in dominant model


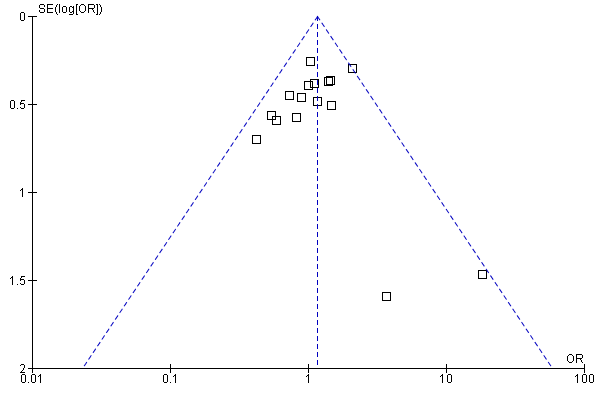


**Figure 10**: Funnel plot of association of *FcγRIIA-131 H/R* polymorphism with idiopathic thrombocytopenic purpura susceptibility in recessive model


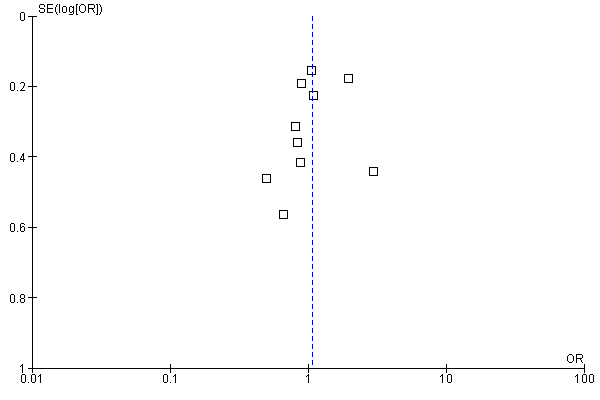


**Figure 11**: Funnel plot of association of *FcγRIIB-232 I/T* polymorphism with idiopathic thrombocytopenic purpura susceptibility in allelic model


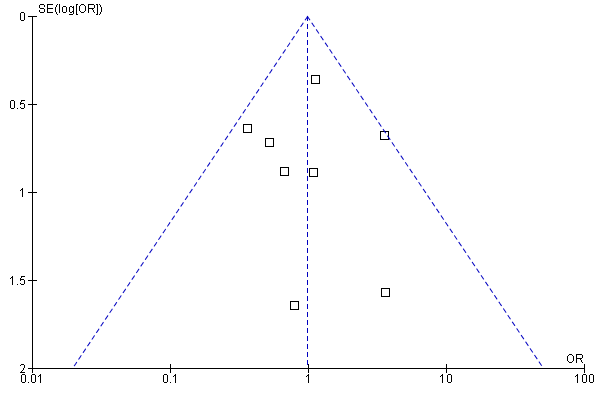


**Figure 12**: Funnel plot of association of *FcγRIIB-232 I/T* polymorphism with idiopathic thrombocytopenic purpura susceptibility in homozygous model


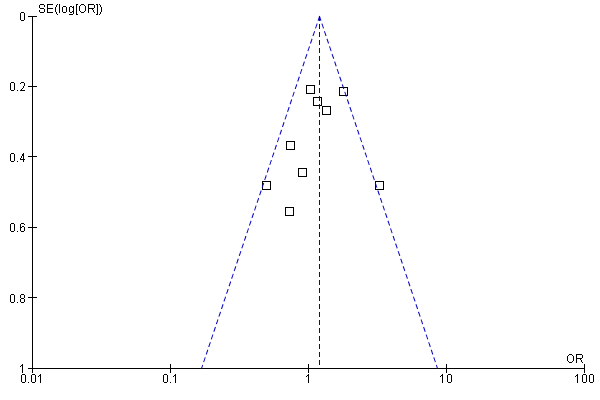


**Figure 13**: Funnel plot of association of *FcγRIIB-232 I/T* polymorphism with idiopathic thrombocytopenic purpura susceptibility in heterozygous model


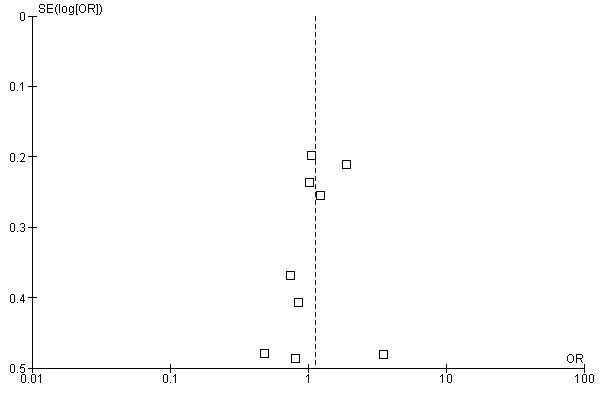


**Figure 14**: Funnel plot of association of *FcγRIIB-232 I/T* polymorphism with idiopathic thrombocytopenic purpura susceptibility in dominant model


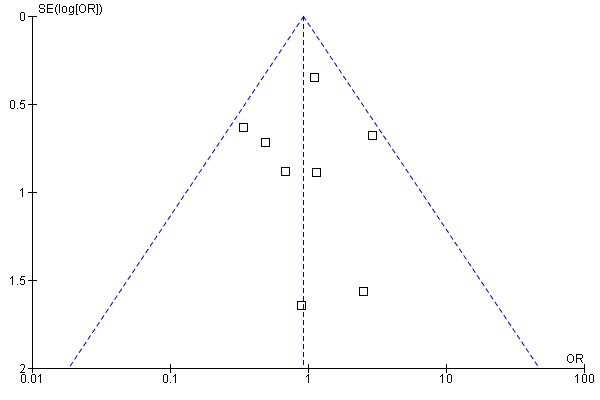


**Figure 15**: Funnel plot of association of *FcγRIIB-232 I/T* polymorphism with idiopathic thrombocytopenic purpura susceptibility in recessive model


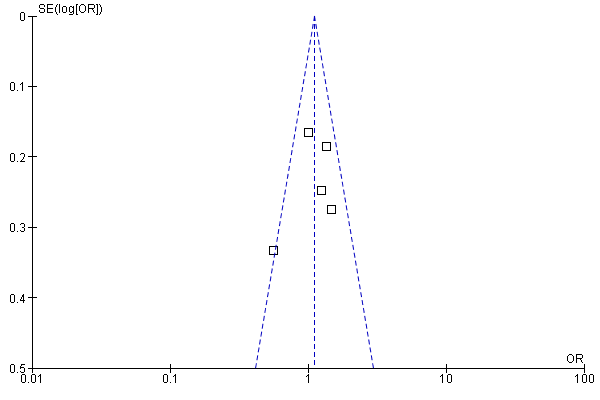


**Figure 16**: Funnel plot of association of *CTLA-4 exon 1 A49G* polymorphism with idiopathic thrombocytopenic purpura susceptibility in allelic model


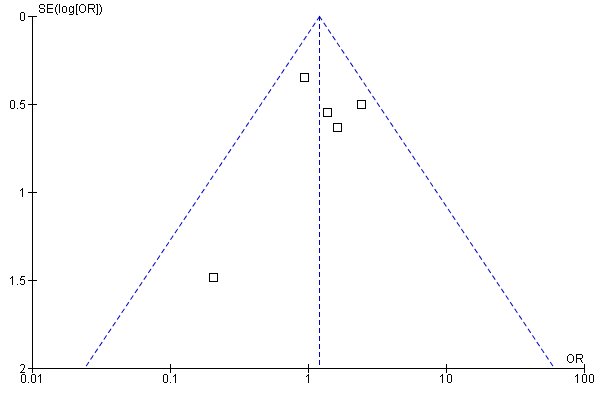


**Figure 17**: Funnel plot of association of *CTLA-4 exon 1 A49G* polymorphism with idiopathic thrombocytopenic purpura susceptibility in homozygous model


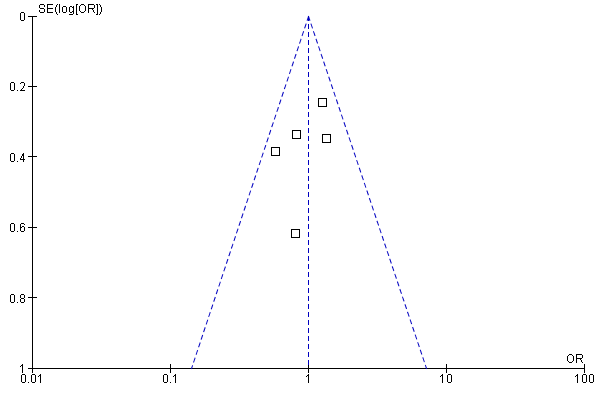


**Figure 18**: Funnel plot of association of *CTLA-4 exon 1 A49G* polymorphism with idiopathic thrombocytopenic purpura susceptibility in heterozygous model


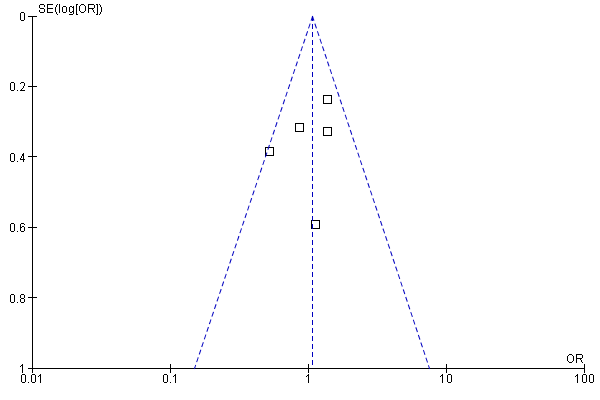


**Figure 19**: Funnel plot of association of *CTLA-4 exon 1 A49G* polymorphism with idiopathic thrombocytopenic purpura susceptibility in dominant model


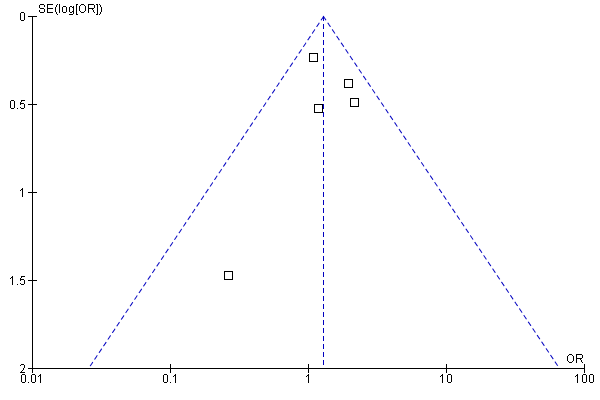


**Figure 20**: Funnel plot of association of *CTLA-4 exon 1 A49G* polymorphism with idiopathic thrombocytopenic purpura susceptibility in recessive model


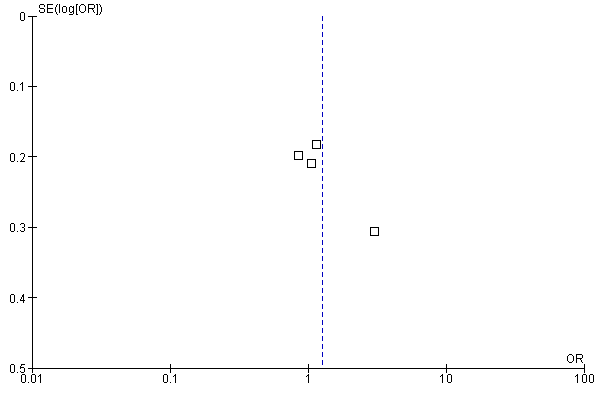


**Figure 21**: Funnel plot of association of *CTLA-4 CT60* polymorphism with idiopathic thrombocytopenic purpura susceptibility in allelic model


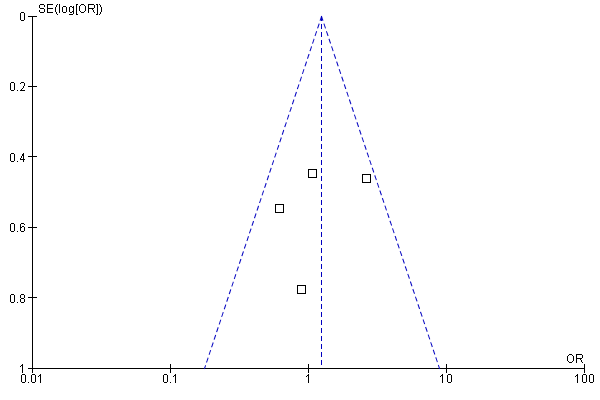


**Figure 22**: Funnel plot of association of *CTLA-4 CT60* polymorphism with idiopathic thrombocytopenic purpura susceptibility in homozygous model


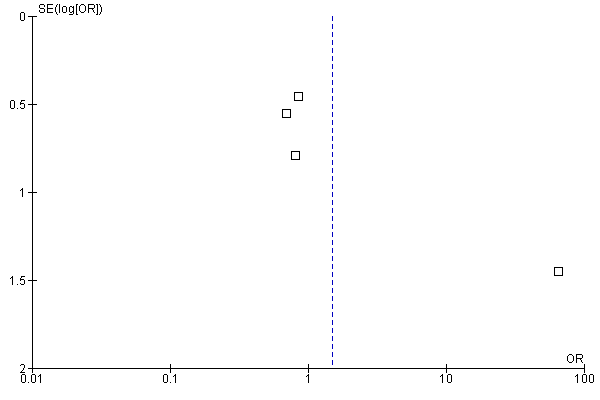


**Figure 23**: Funnel plot of association of *CTLA-4 CT60* polymorphism with idiopathic thrombocytopenic purpura susceptibility in heterozygous model


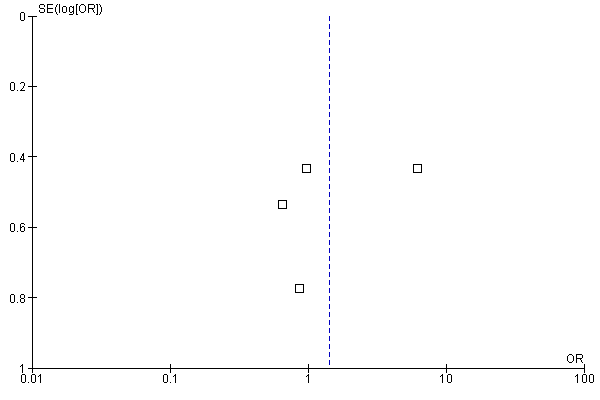


**Figure 24**: Funnel plot of association of *CTLA-4 CT60* polymorphism with idiopathic thrombocytopenic purpura susceptibility in dominant model


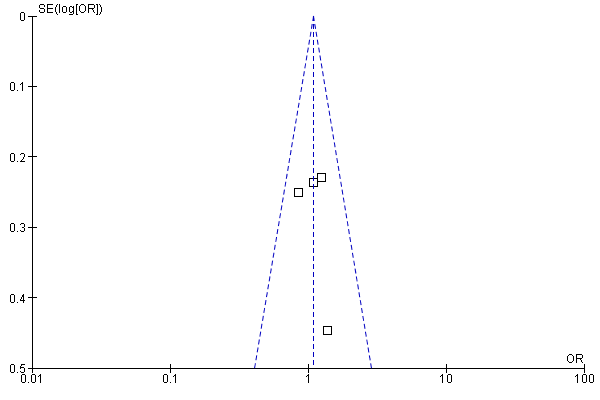


**Figure 25**: Funnel plot of association of *CTLA-4 CT60* polymorphism with idiopathic thrombocytopenic purpura susceptibility in recessive model

**
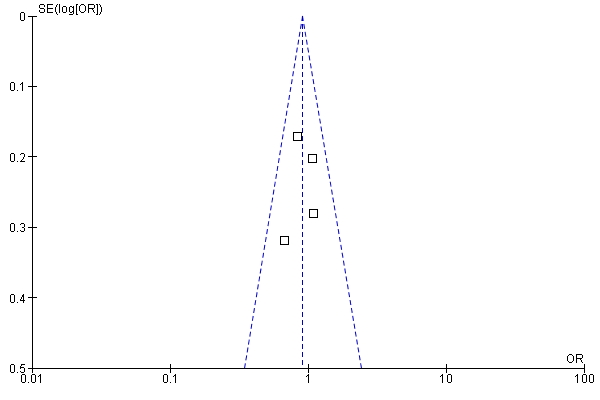
**

**Figure 26**: Funnel plot of association of *CD40 rs4810485* polymorphism with idiopathic thrombocytopenic purpura susceptibility in allelic model


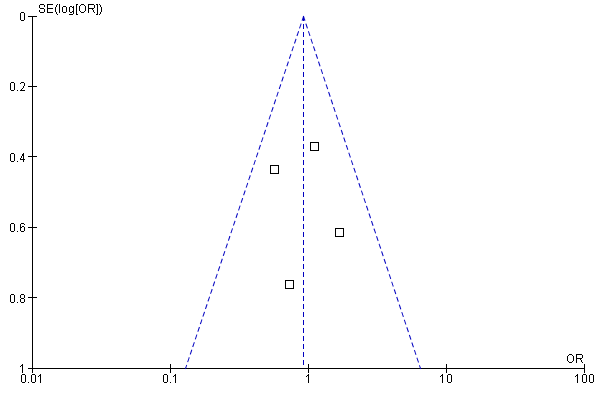


**Figure 27**: Funnel plot of association of *CD40 rs4810485* polymorphism with idiopathic thrombocytopenic purpura susceptibility in homozygous model


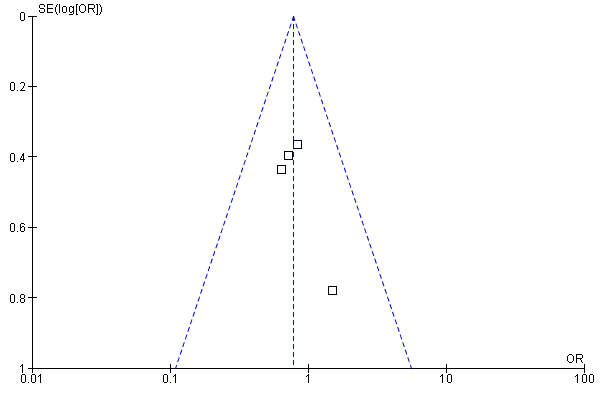


**Figure 28**: Funnel plot of association of *CD40 rs4810485* polymorphism with idiopathic thrombocytopenic purpura susceptibility in heterozygous model


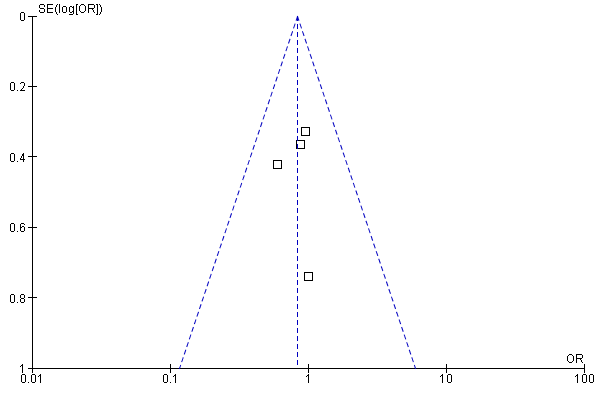


**Figure 29**: Funnel plot of association of *CD40 rs4810485* polymorphism with idiopathic thrombocytopenic purpura susceptibility in dominant model


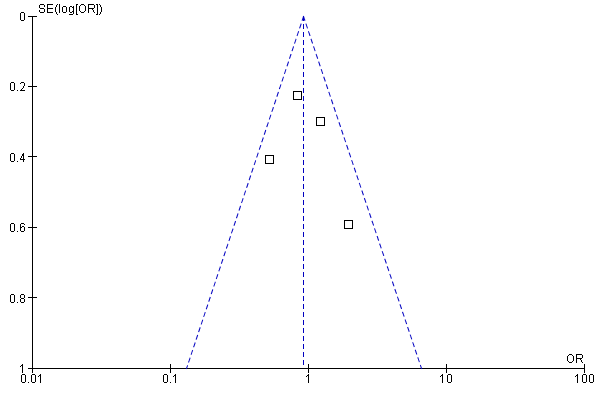


**Figure 30**: Funnel plot of association of *CD40 rs4810485* polymorphism with idiopathic thrombocytopenic purpura susceptibility in recessive model


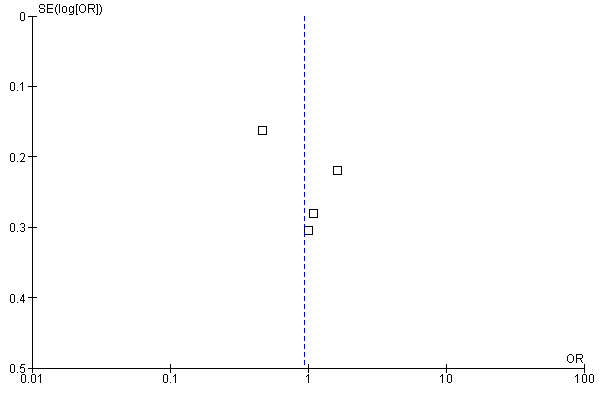


**Figure 31**: Funnel plot of association of *CD40 rs1883832* polymorphism with idiopathic thrombocytopenic purpura susceptibility in allelic model


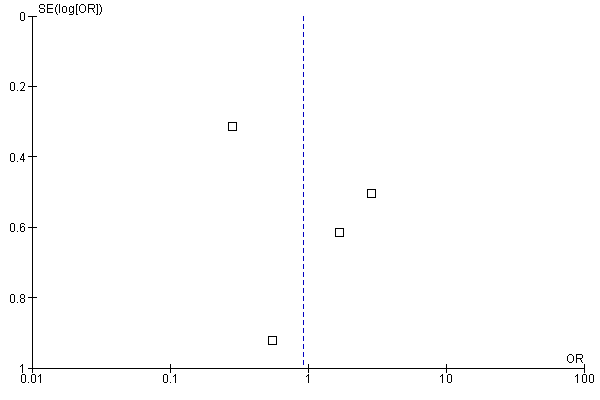


**Figure 32**: Funnel plot of association of *CD40 rs1883832* polymorphism with idiopathic thrombocytopenic purpura susceptibility in homozygous model


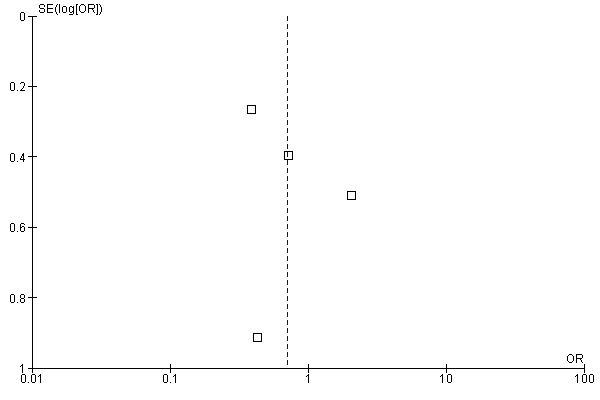


**Figure 33**: Funnel plot of association of *CD40 rs1883832* polymorphism with idiopathic thrombocytopenic purpura susceptibility in heterozygous model


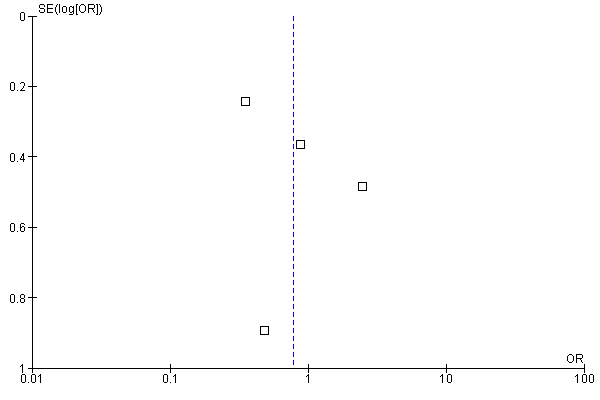


**Figure 34**: Funnel plot of association of *CD40 rs1883832* polymorphism with idiopathic thrombocytopenic purpura susceptibility in dominant model


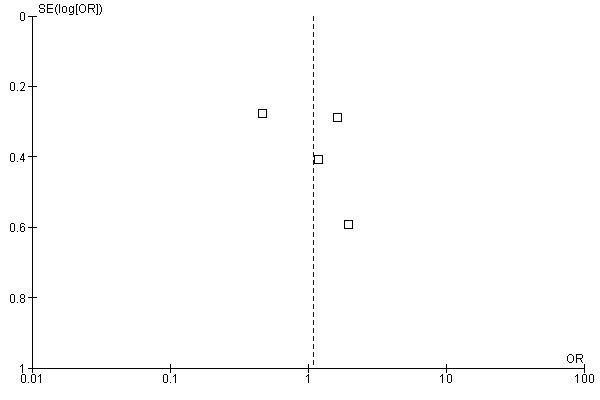


**Figure 35**: Funnel plot of association of *CD40 rs1883832* polymorphism with idiopathic thrombocytopenic purpura susceptibility in recessive model
