# Supplementary material for: Evaluation of CD16, CD32, CD40, and CD152 polymorphisms in immune thrombocytopenia patients: a systematic review, meta-analysis, and trial sequential analysis
Source: Front Med (Lausanne). 2026 Jun 23;13:1777678. doi: 10.3389/fmed.2026.1777678 (PMC13337454; doi:10.3389/fmed.2026.1777678)
Supplement: Supplementary file 3 [file Supplementary_file_3.docx]

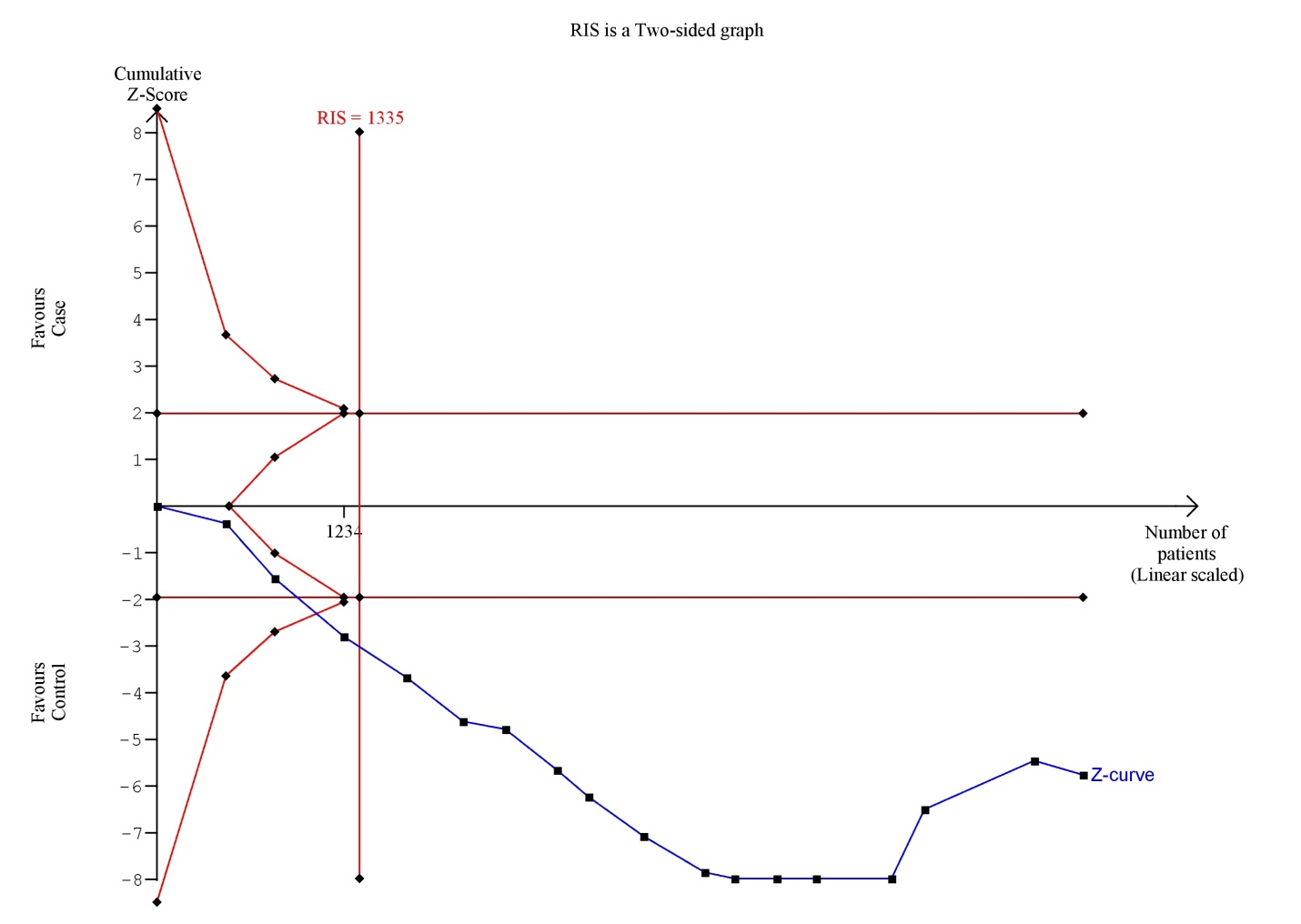


**Figure 1**: Trial sequential analysis of association of *FcγRIIIA-158 F/V* polymorphism with idiopathic thrombocytopenic purpura susceptibility in allelic model


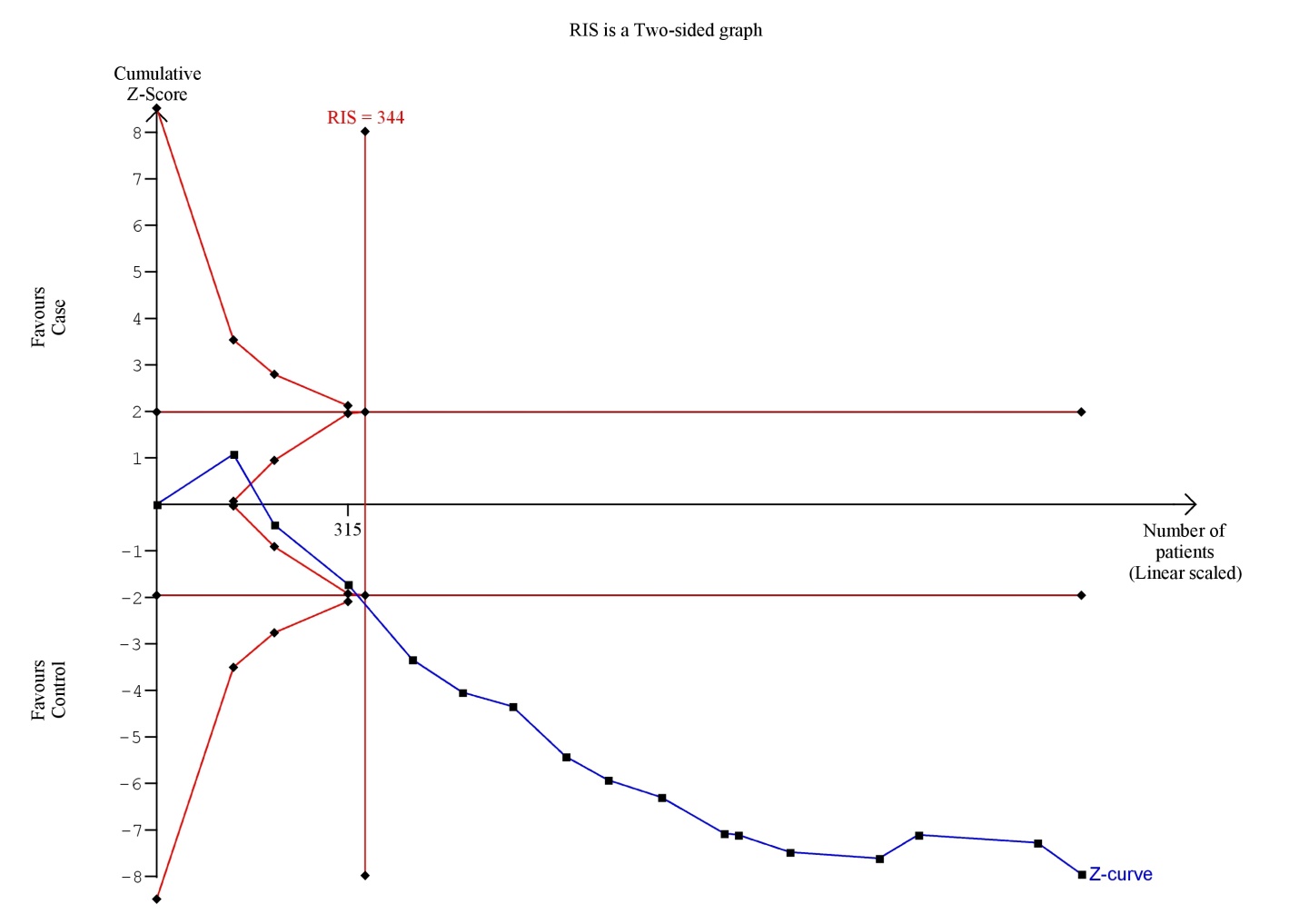


**Figure 2**: Trial sequential analysis of association of *FcγRIIIA-158 F/V* polymorphism with idiopathic thrombocytopenic purpura susceptibility in homozygous model


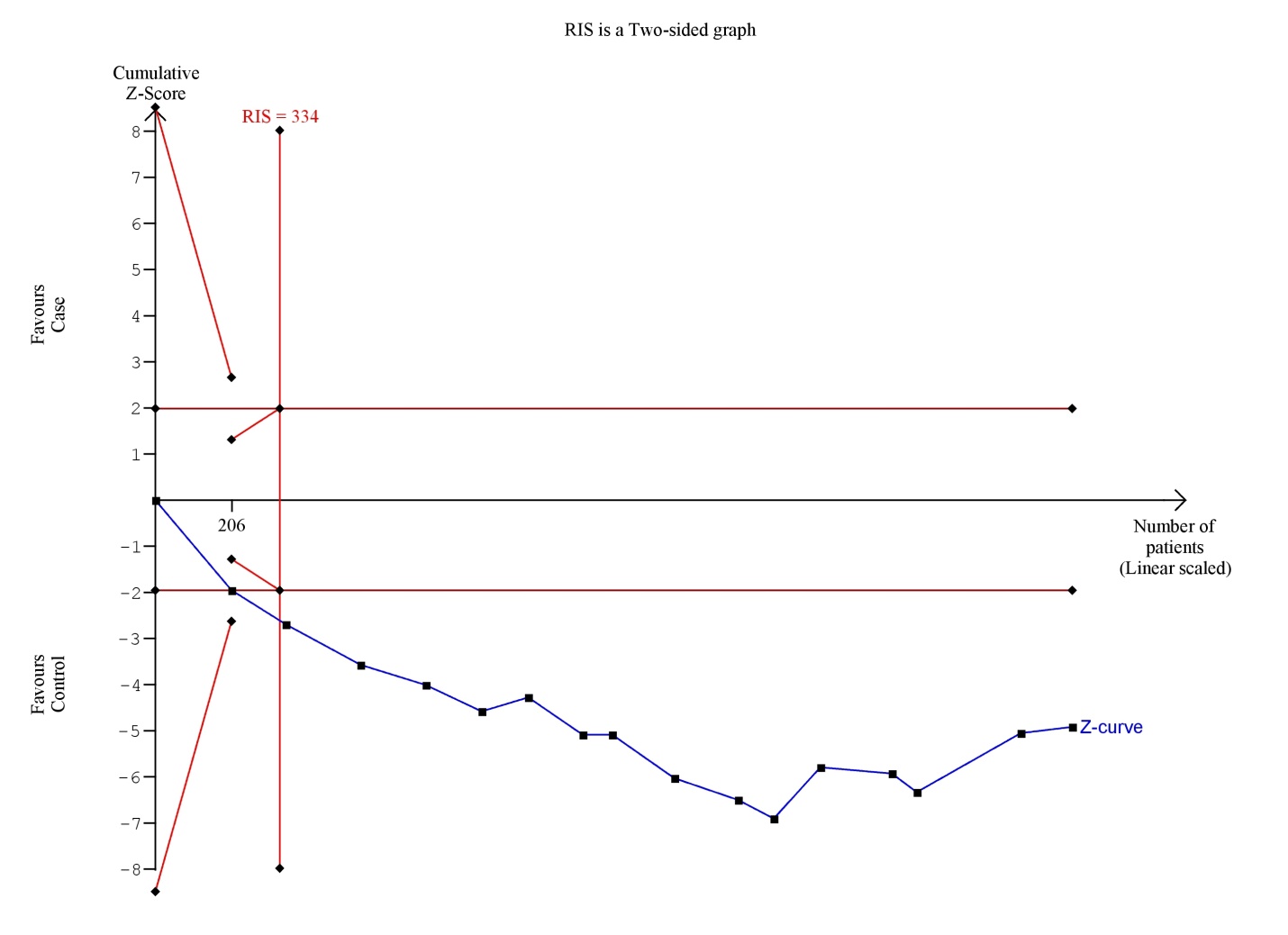


**Figure 3**: Trial sequential analysis of association of *FcγRIIIA-158 F/V* polymorphism with idiopathic thrombocytopenic purpura susceptibility in heterozygous model


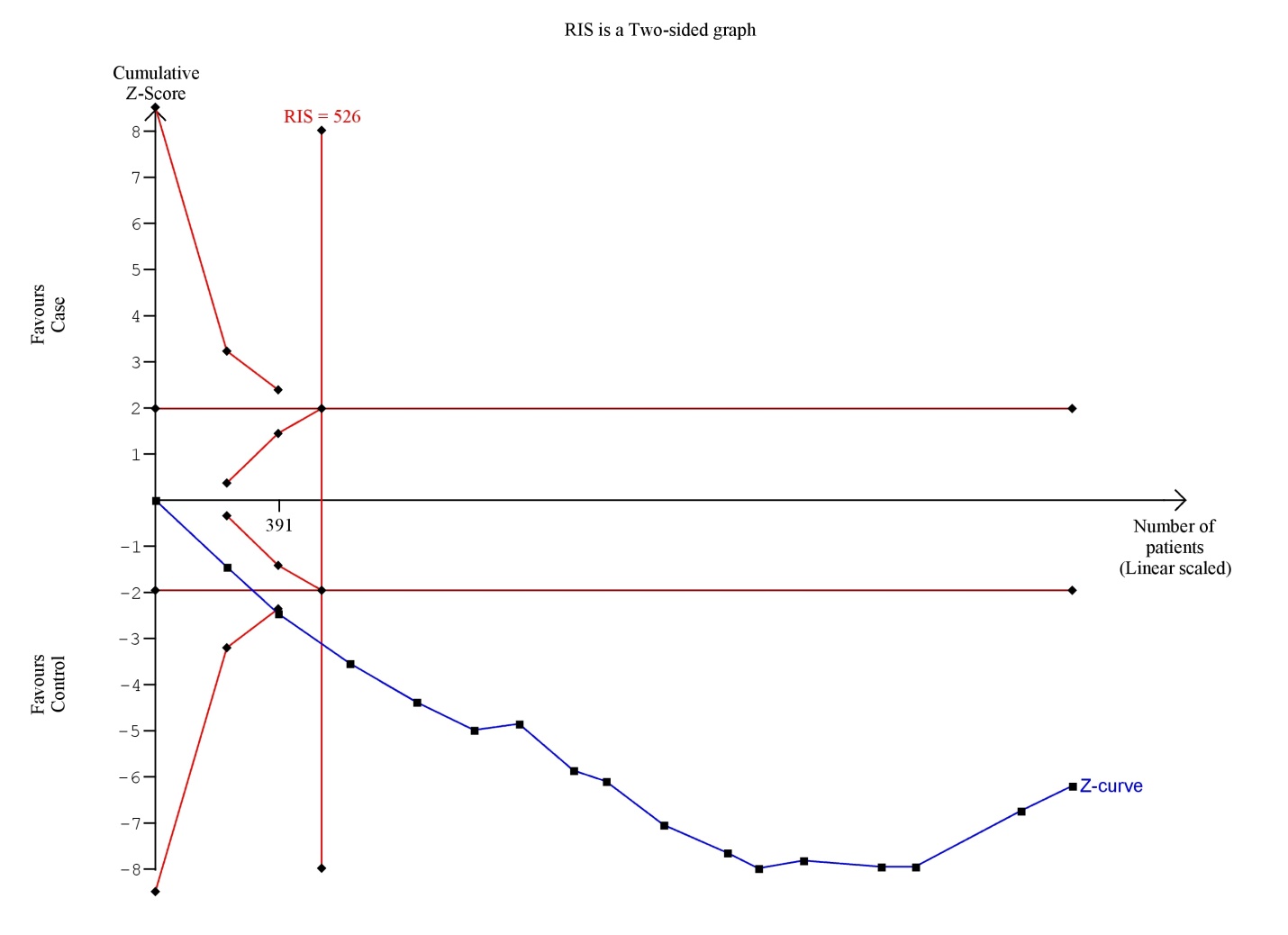


**Figure 4**: Trial sequential analysis of association of *FcγRIIIA-158 F/V* polymorphism with idiopathic thrombocytopenic purpura susceptibility in dominant model


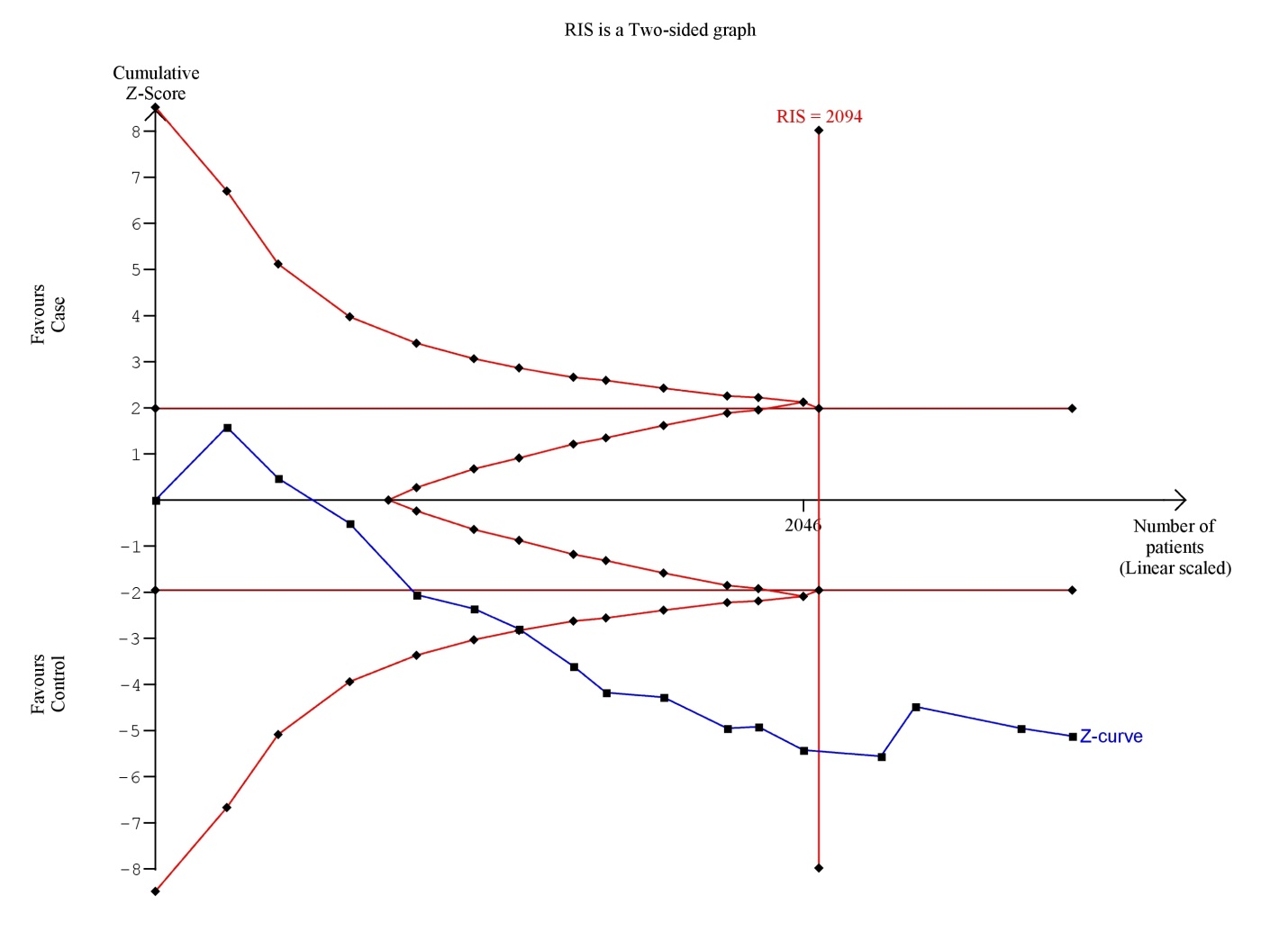


**Figure 5**: Trial sequential analysis of association of *FcγRIIIA-158 F/V* polymorphism with idiopathic thrombocytopenic purpura susceptibility in recessive model


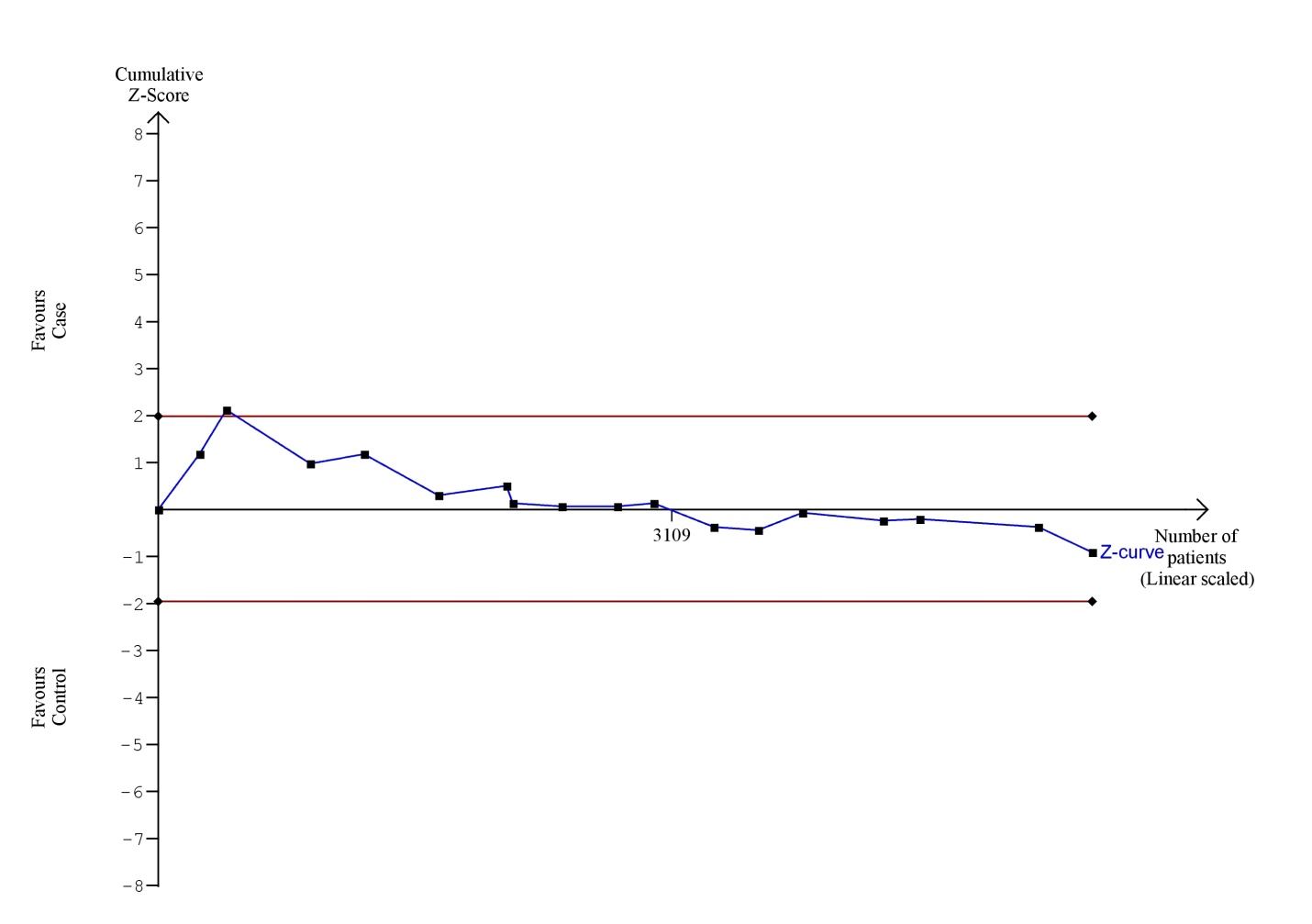


**Figure 6**: Trial sequential analysis of association of *FcγRIIA-131 H/R* polymorphism with idiopathic thrombocytopenic purpura susceptibility in allelic model


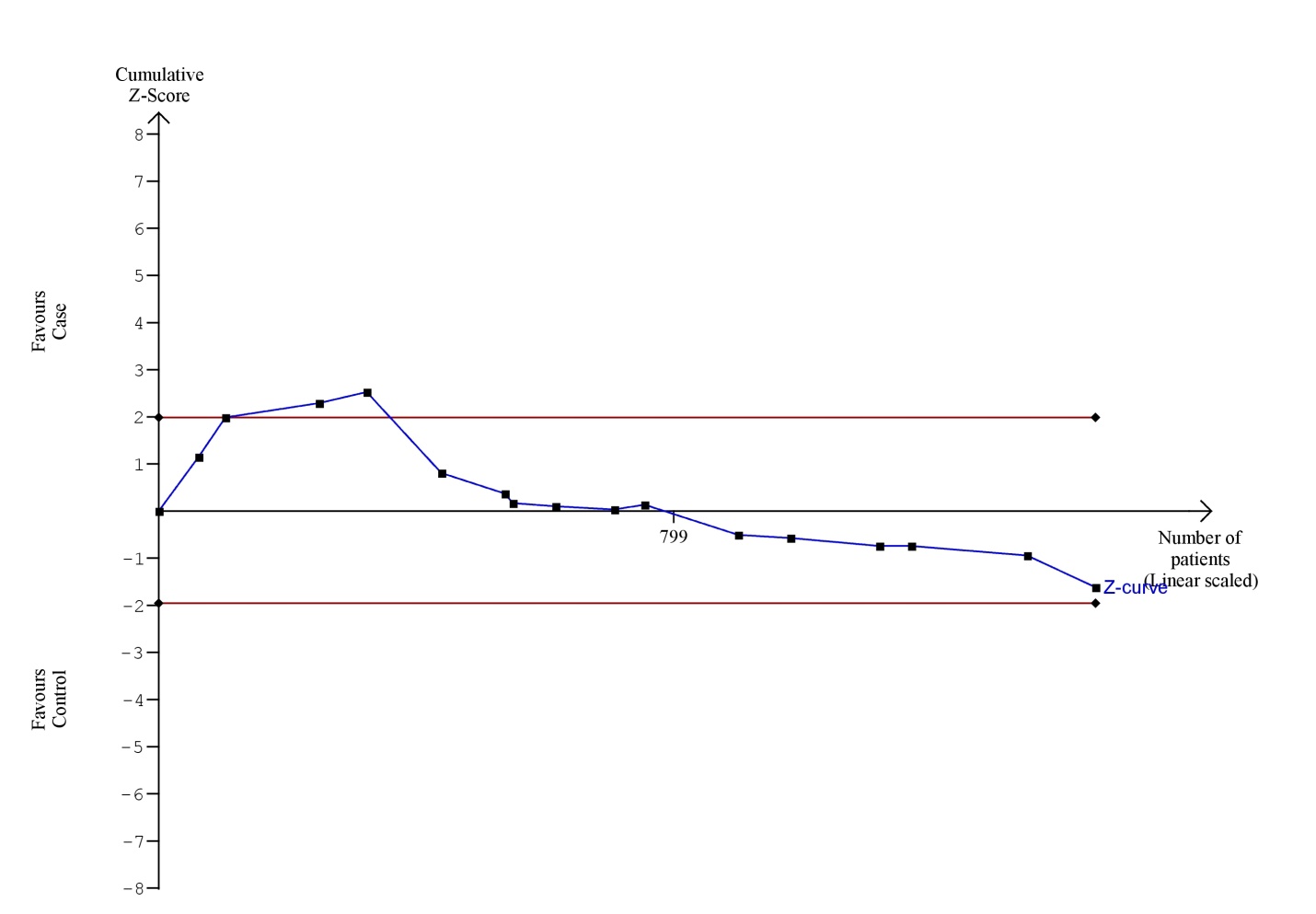


**Figure 7**: Trial sequential analysis of association of *FcγRIIA-131 H/R* polymorphism with idiopathic thrombocytopenic purpura susceptibility in homozygous model


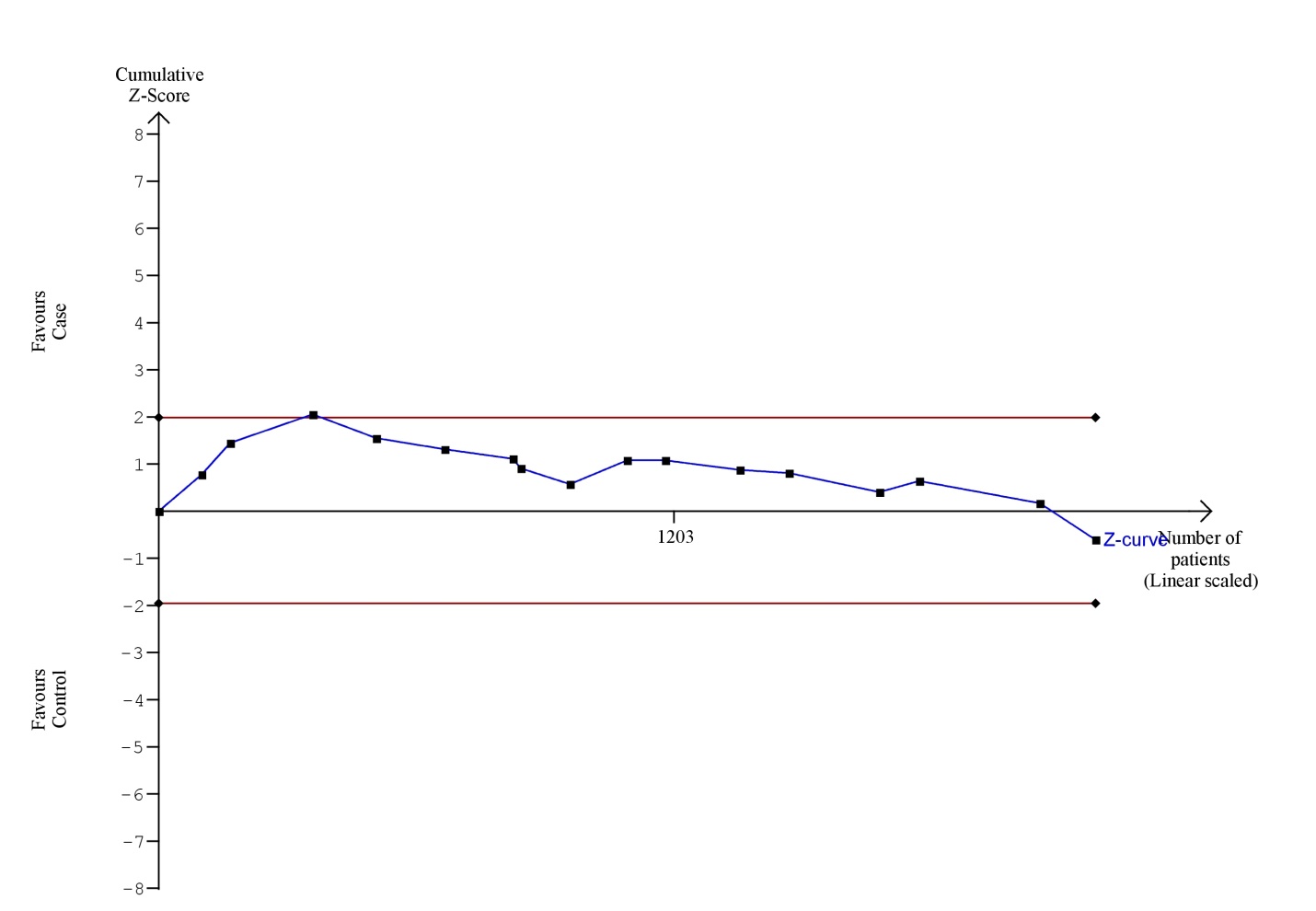


**Figure 8**: Trial sequential analysis of association of *FcγRIIA-131 H/R* polymorphism with idiopathic thrombocytopenic purpura susceptibility in heterozygous model


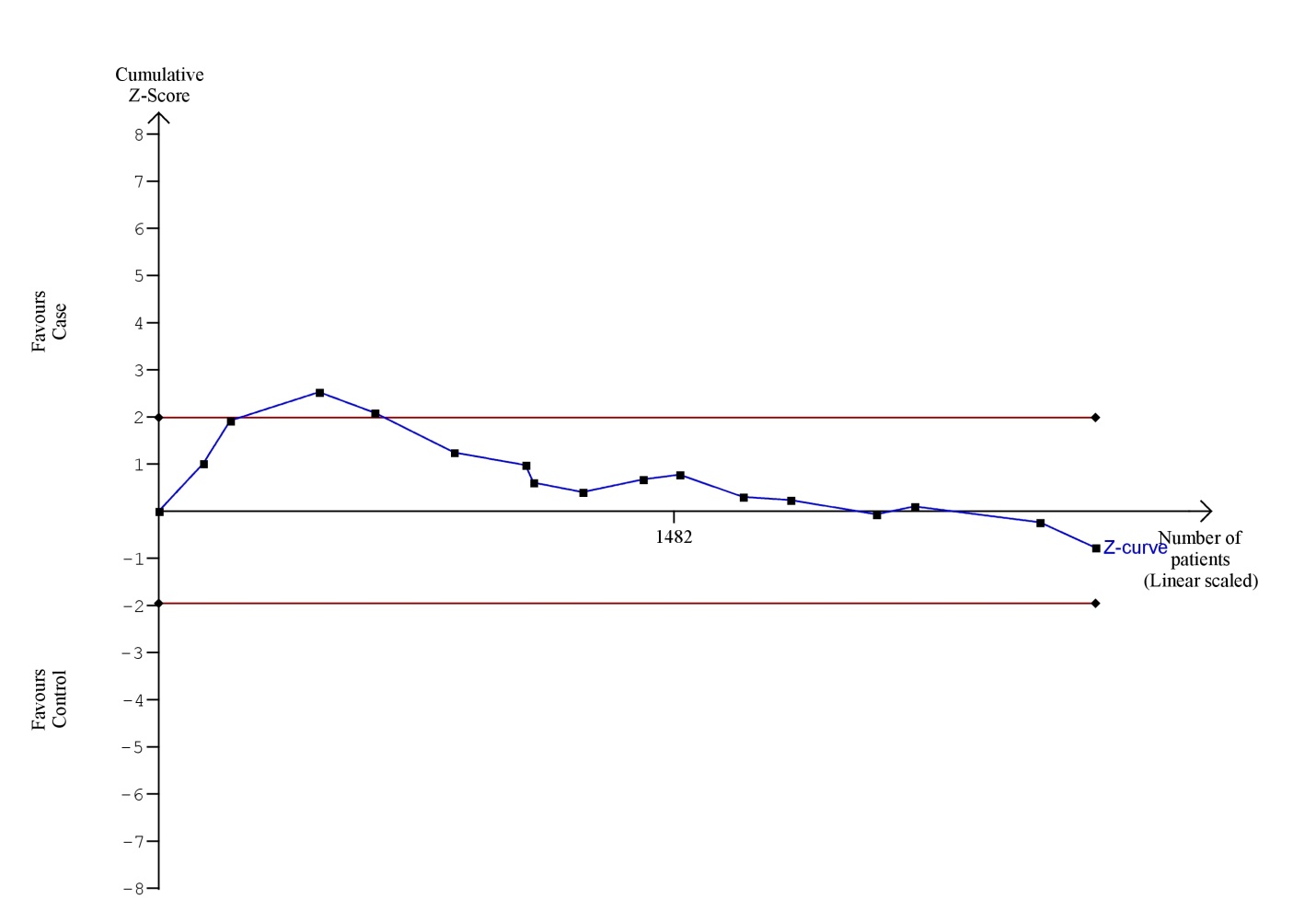


**Figure 9**: Trial sequential analysis of association of *FcγRIIA-131 H/R* polymorphism with idiopathic thrombocytopenic purpura susceptibility in dominant model


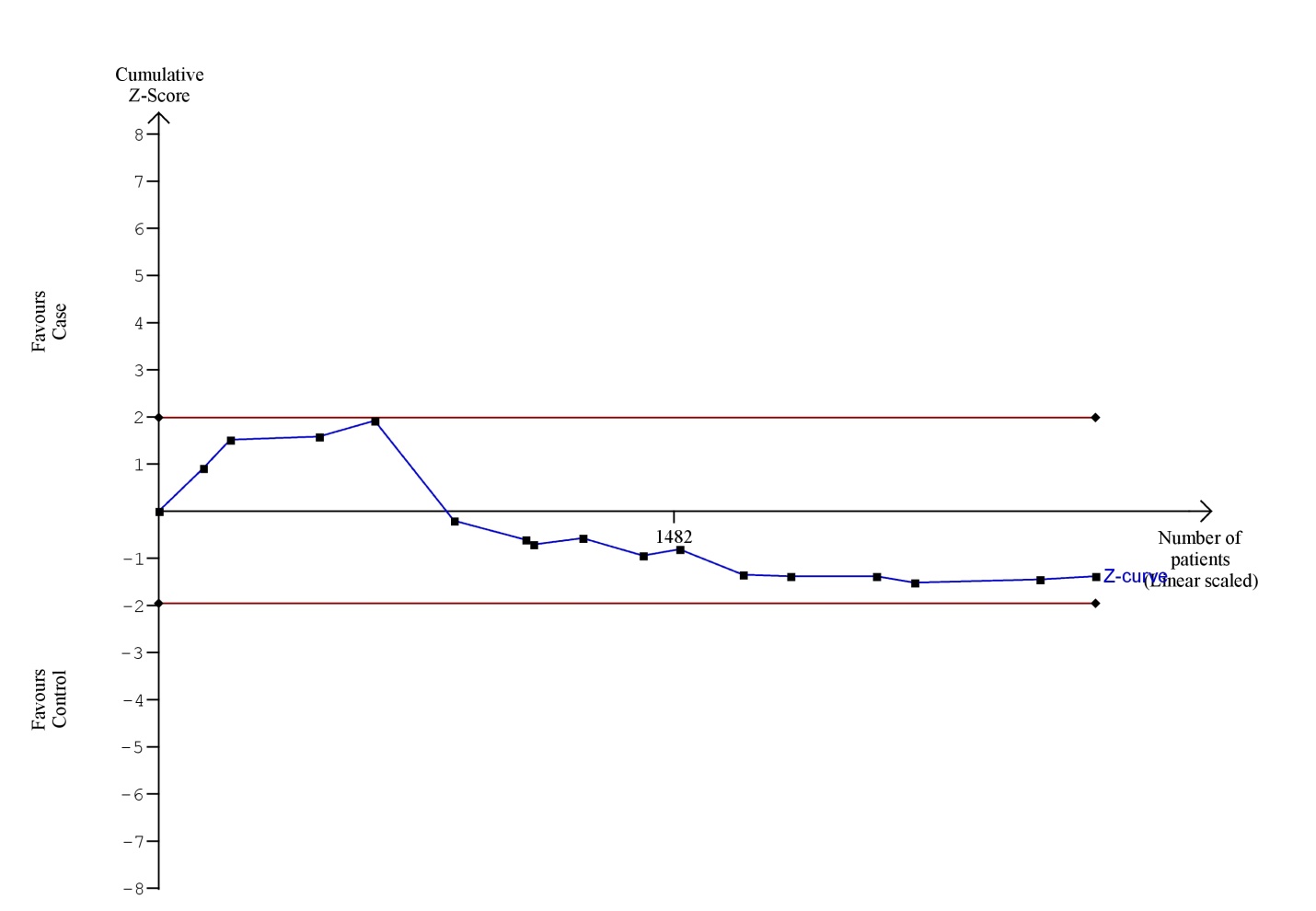


**Figure 10**: Trial sequential analysis of association of *FcγRIIA-131 H/R* polymorphism with idiopathic thrombocytopenic purpura susceptibility in recessive model


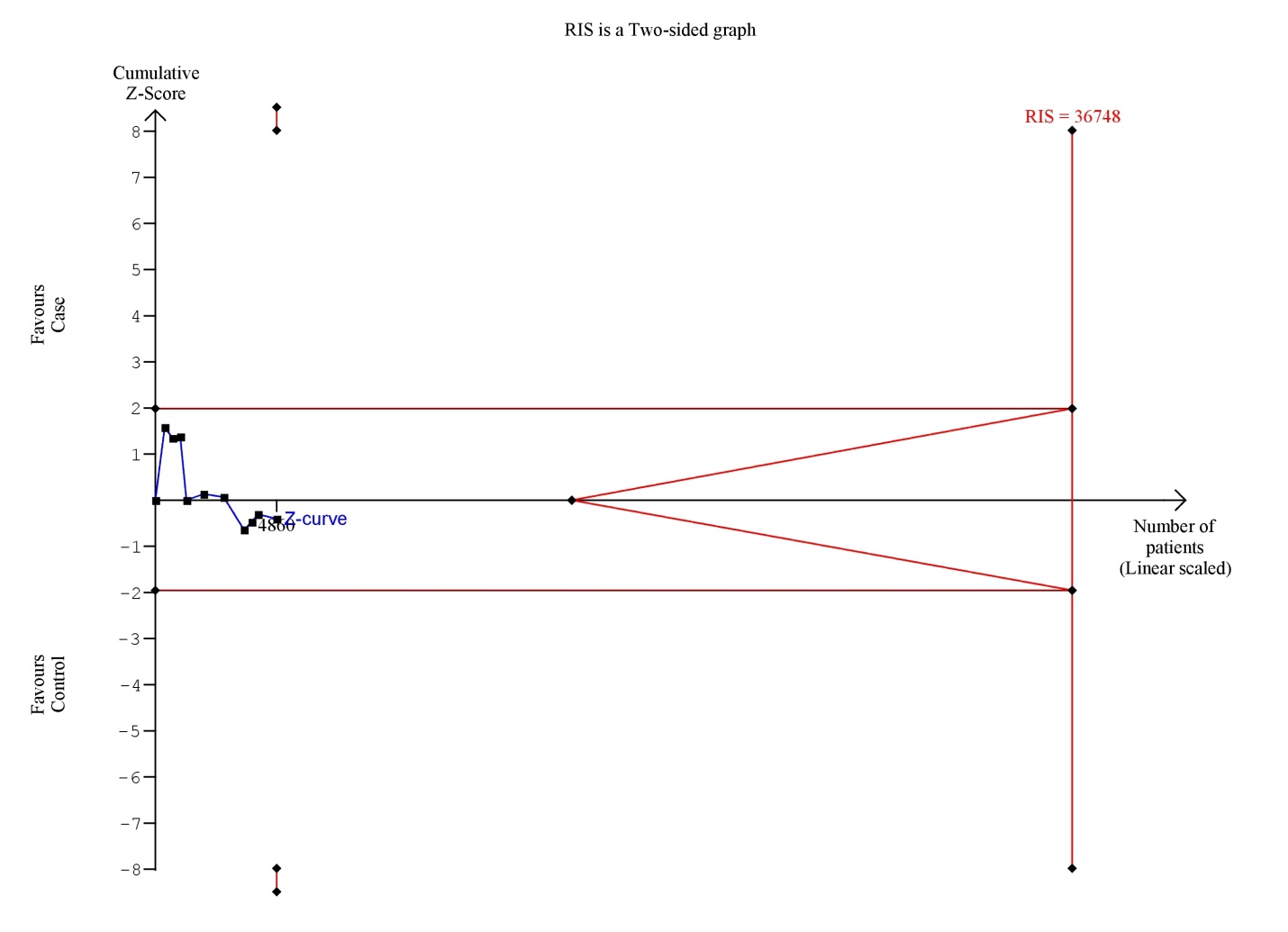


**Figure 11**: Trial sequential analysis of association of *FcγRIIB-232 I/T* polymorphism with idiopathic thrombocytopenic purpura susceptibility in allelic model


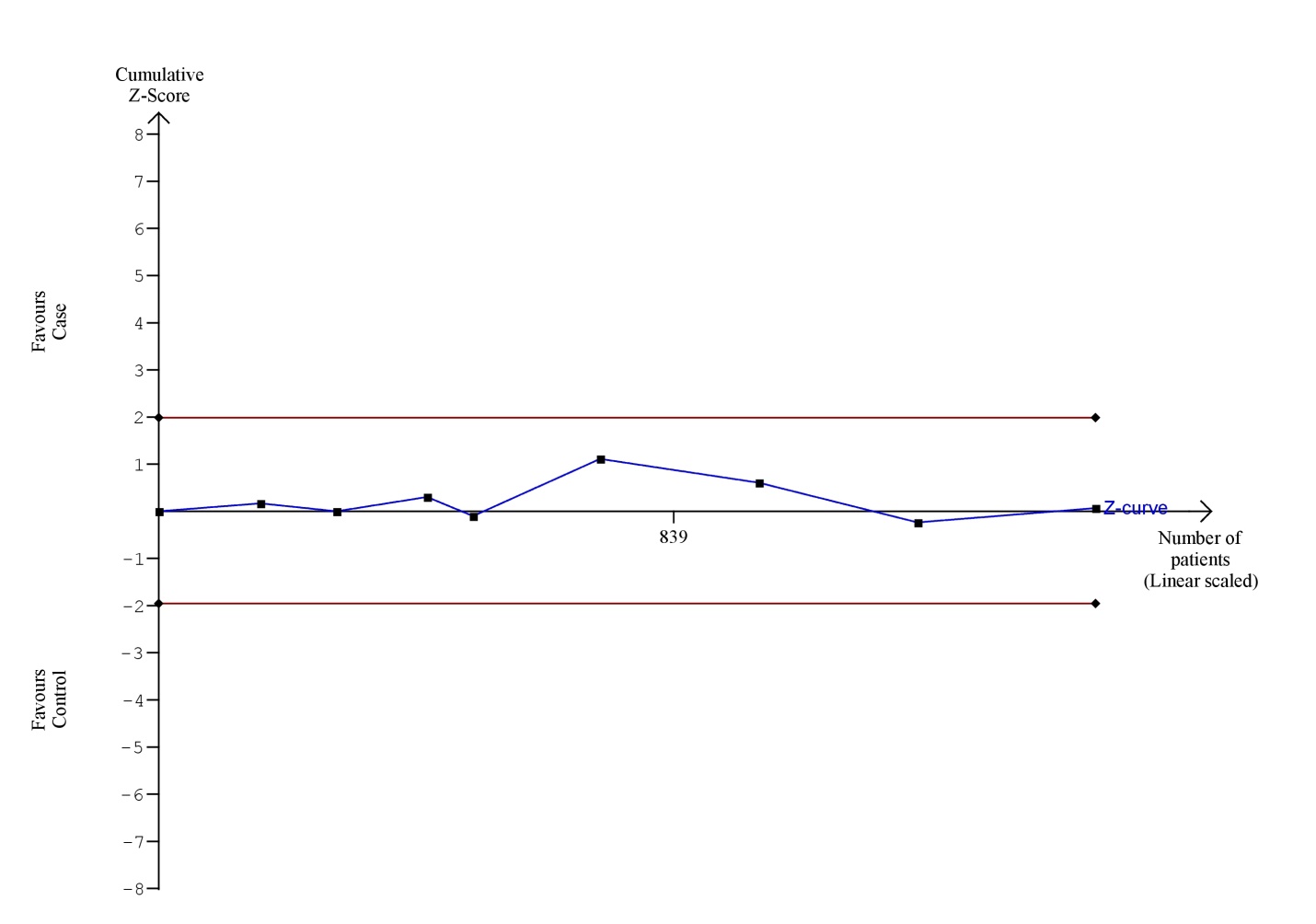


**Figure 12**: Trial sequential analysis of association of *FcγRIIB-232 I/T* polymorphism with idiopathic thrombocytopenic purpura susceptibility in homozygous model


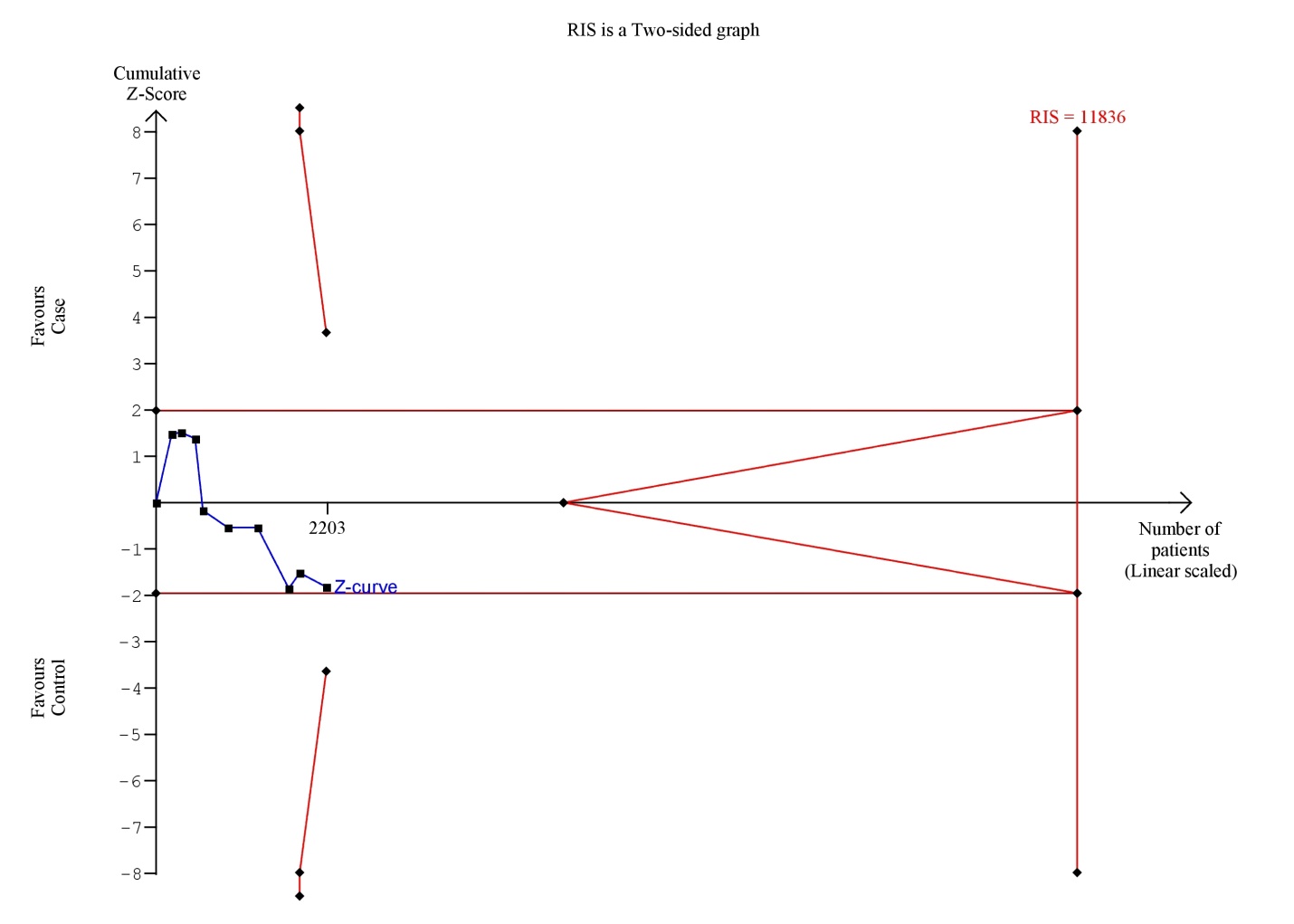


**Figure 13**: Trial sequential analysis of association of *FcγRIIB-232 I/T* polymorphism with idiopathic thrombocytopenic purpura susceptibility in heterozygous model


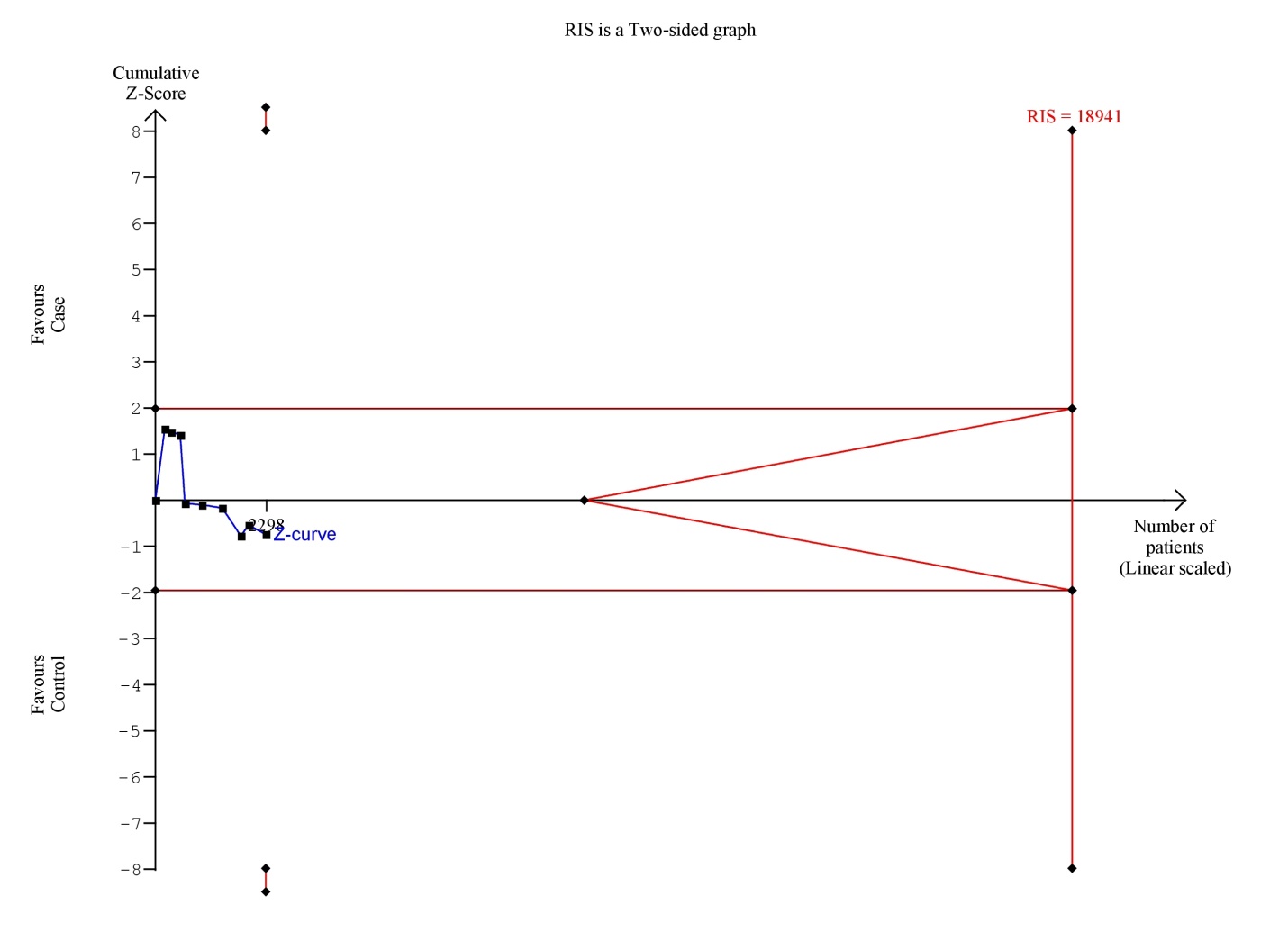


**Figure 14**: Trial sequential analysis of association of *FcγRIIB-232 I/T* polymorphism with idiopathic thrombocytopenic purpura susceptibility in dominant model


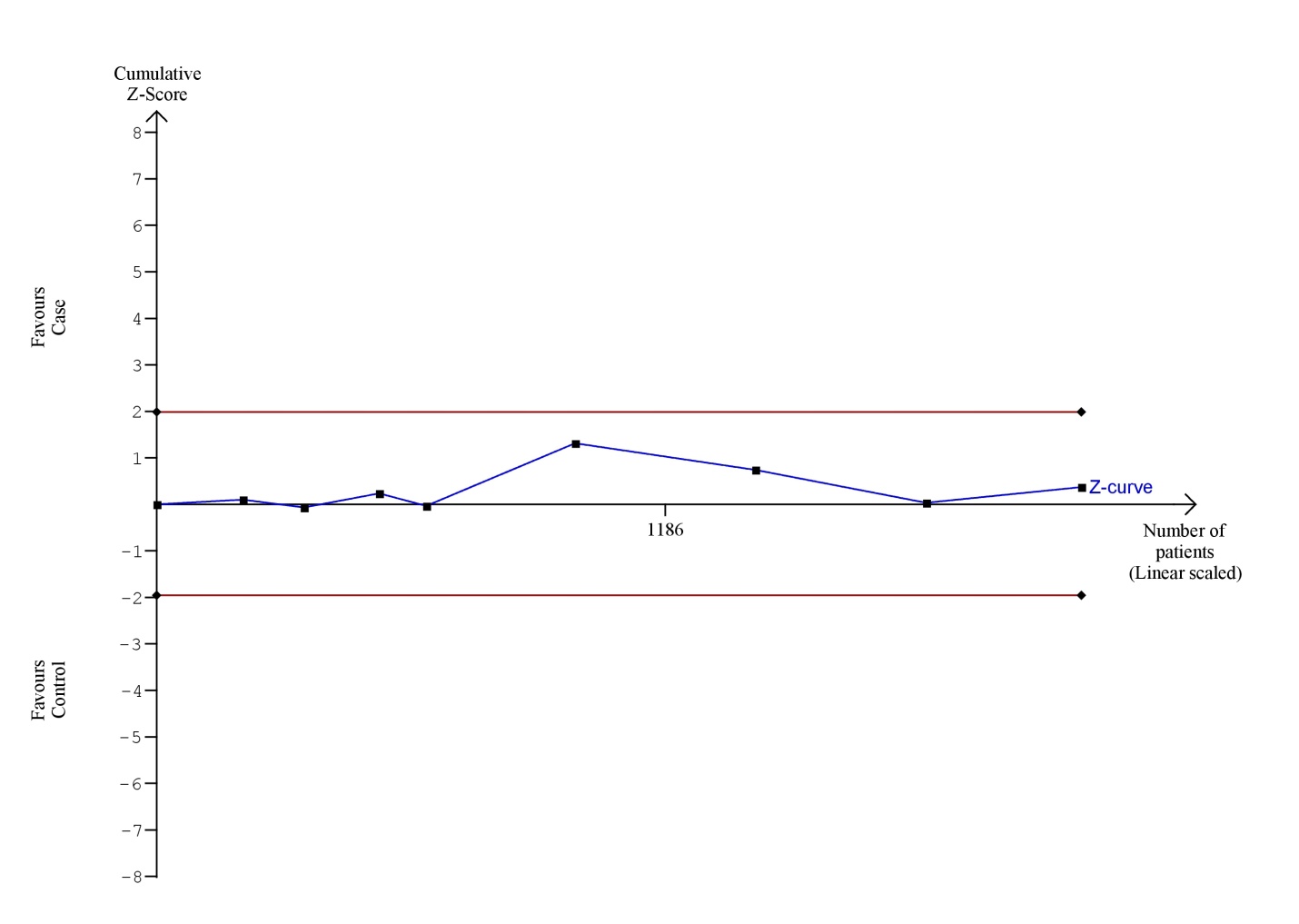


**Figure 15**: Trial sequential analysis of association of *FcγRIIB-232 I/T* polymorphism with idiopathic thrombocytopenic purpura susceptibility in recessive model
